# Modeling a unit cell: crystallographic refinement procedure using the biomolecular MD simulation platform *Amber*

**DOI:** 10.1107/S2052252521011891

**Published:** 2021-12-16

**Authors:** Oleg Mikhailovskii, Yi Xue, Nikolai R. Skrynnikov

**Affiliations:** a Laboratory of Biomolecular NMR, St Petersburg State University, St Petersburg 199034, Russian Federation; b Department of Chemistry, Purdue University, West Lafayette, IN 47907, USA; c School of Life Sciences, Tsinghua University, Beijing 100084, People’s Republic of China; d Beijing Advanced Innovation Center for Structural Biology, Tsinghua University, Beijing 100084, People’s Republic of China; e Tsinghua University–Peking University Joint Center for Life Sciences, Tsinghua University, Beijing 100084, People’s Republic of China

**Keywords:** protein structure refinement, *Amber*, molecular dynamics, intracrystalline water, ensemble model, maximum likelihood, molecular replacement, *R*_free_, *Phenix*, X-ray crystallography, protein structure determination, computational modeling, restrained simulations, protein crystals

## Abstract

A procedure has been developed for the refinement of crystallographic protein structures based on the biomolecular simulation program *Amber*. The procedure constructs a model representing a crystal unit cell, which generally contains multiple protein molecules and is fully hydrated with TIP3P water. Periodic boundary conditions are applied to the cell in order to emulate the crystal lattice. The refinement is conducted in the form of a specially designed short molecular-dynamics run controlled by the Amber ff14SB force field and the maximum-likelihood potential that encodes the structure-factor-based restraints. The new *Amber*-based refinement procedure has been tested on a set of 84 protein structures. In most cases, the new procedure led to appreciably lower *R*_free_ values compared with those reported in the original PDB depositions or obtained by means of the industry-standard *phenix.refine* program. In particular, the new method has the edge in refining low-accuracy scrambled models. It has also been successful in refining a number of molecular-replacement models, including one with an r.m.s.d. of 2.15 Å. In addition, *Amber*-refined structures consistently show superior *MolProbity* scores. The new approach offers a highly realistic representation of protein–protein interactions in the crystal, as well as of protein–water interactions. It also offers a realistic representation of protein crystal dynamics (akin to ensemble-refinement schemes). Importantly, the method fully utilizes the information from the available diffraction data, while relying on state-of-the-art molecular-dynamics modeling to assist with those elements of the structure that do not diffract well (for example mobile loops or side chains). Finally, it should be noted that the protocol employs no tunable parameters, and the calculations can be conducted in a matter of several hours on desktop computers equipped with graphical processing units or using a designated web service.

## Abstract 2

Based on the molecular-dynamics simulation platform *Amber*, a refinement algorithm has been developed to obtain structural models of protein crystals in the form of a fully hydrated unit cell. These models typically yield better *R*_free_ values and *MolProbity* scores compared with the PDB-deposited or *Phenix*-refined coordinates. The new algorithm has a favorable radius of convergence, is simple to use and is fast.

## 1. Introduction

The contemporary refinement of macromolecular structures is an interactive progression that is an interplay between model building, automated model optimization, validation and visual confirmation of the agreement between model and electron-density maps. This interactive process may include manual model adjustments. In addition to coordinates, the refinement involves other variables, such as atomic displacement parameters, anisotropic scaling matrices, anomalous scattering factors *etc*. Multiple programs that are used to refine bio­molecular structures can interface with each other, with many of them being integrated into the large software suites *Phenix* (Liebschner *et al.*, 2019 ▸) and *CCP*4 (Winn *et al.*, 2011 ▸). Structure refinement improves the accuracy of protein structures, which is particularly useful, for example, for quantum-chemical investigation of enzymatic catalysis (Friesner & Guallar, 2005 ▸) or rational drug design (Blundell, 2017 ▸). Higher-quality protein coordinates also benefit the parameterization of various knowledge-based force fields and the training of structure-prediction algorithms (Jumper *et al.*, 2021 ▸; Xu & Zhang, 2012 ▸). Importantly, there are many examples of how post-deposition refinement leads to improved or even novel biological insights (Touw *et al.*, 2016 ▸).

In this report, we focus on the step of automated structure refinement, which is normally performed by *phenix.refine* (Afonine *et al.*, 2012 ▸) or the *REFMAC* module (Murshudov *et al.*, 2011 ▸) within *CCP*4. This step involves small adjustments of atomic coordinates guided by experimental structure factors and by knowledge-based stereochemical restraints. The need for stereochemical restraints arises from the limited crystallographic resolution; the restraints serve to regularize bond lengths, bond angles, planarity and improper angles, thus improving the overall quality of the structure. Originally, stereochemical restraints were parameterized based on small-molecule structures in the Cambridge database (Engh & Huber, 2001 ▸). Eventually, more sophisticated parameterizations were developed, for example the conformation-dependent library (CDL), which accounts for the dependence of the bond geometry on the conformation of the peptide chain (Tronrud *et al.*, 2010 ▸). This parameterization has been included as a default choice in the refinement module of *Phenix* (Moriarty *et al.*, 2016 ▸). Additional restraints have been used to avoid steric clashes between atoms; in the case of *Phenix*, these are implemented in the form of a simple repulsive potential (Afonine *et al.*, 2018 ▸).

It was recognized early on (Brunger *et al.*, 1989 ▸) that the body of restraints used in crystallographic refinement can be viewed as a special case of a force field (FF), similar to the force fields that are used in molecular modeling. In the existing refinement protocols, the target function is comprised of two terms, 

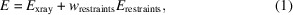

where *E*_xray_ reflects the difference between the calculated and experimental structure factors (SFs), *E*_restraints_ reflects the difference between the current and idealized geometry, and the weight *w*_restraints_ regulates the relative influence of these two terms. It is also possible to set up the refinement procedure using molecular-dynamics (MD) software, where the same expression can be rewritten as

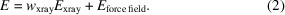




Here, the geometry restraints are packaged into the force-field potential *E*_force field_, the SF-based penalty function is treated as the ancillary potential *E*_xray_ and the relative weight *w*_xray_ is attached to the latter term.

Given this dual perspective, there are two main lines of development of crystallographic refinement procedures.

Firstly, in the context of equation (1), the target function *E*_restraints_ can be further improved and expanded. For instance, *phenix.refine* (Afonine *et al.*, 2012 ▸) has options for noncrystallographic symmetry restraints (enforcing the similarity between protein molecules in the crystal asymmetric unit), Ramachandran map restraints (utilizing empirical knowledge about the conformational preferences of peptide chains) and many other similar extensions (Afonine *et al.*, 2018 ▸). Furthermore, new classes of restraints have been introduced that exploit experimental data other than X-ray diffraction data. In particular, *phenix.refine* has been adapted to handle neutron diffraction data (Afonine *et al.*, 2010 ▸), while the refinement module *REFMAC*5 (Murshudov *et al.*, 2011 ▸) from another leading software package, *CCP*4, has been extended to include solution NMR data (Rinaldelli *et al.*, 2014 ▸).

Secondly, in the context of equation (2), one can use one of the existing state-of-the-art biomolecular force fields to represent *E*_force field_. Such advanced force fields should not only take care of covalent geometry, but should also help to improve the coordinates of mobile side chains, loops and terminal segments (tails), which are poorly defined in the electron-density map. This line of research was pioneered by Brunger and Karplus (Brunger *et al.*, 1987 ▸), resulting in the program *X-PLOR* (Brunger, 1990 ▸). This program gives the user a choice of force fields, which includes CHARMM, Amber and Amber/OPLS (Mackerell, 2004 ▸) along with more specialized variants built around stereochemical restraints. *X-PLOR* has seen considerable use in the context of crystallographic refinement, but the choice of force field is hardly ever mentioned in the resulting publications (Moore *et al.*, 1997 ▸; Rutenber *et al.*, 1991 ▸).

Two decades later, Schnieders, Fenn, Brunger and Pande demonstrated the refinement of crystallographic protein structures using the polarizable force field AMOEBA (Fenn *et al.*, 2010 ▸), which offers a particularly good representation of electrostatics. The scheme was soon adapted for computations on graphical processors (GPUs) using the specially developed MD engine *FFX* (Schnieders *et al.*, 2011 ▸). Despite its successful initial demonstration, this approach has only been used to refine a few structures (Andrews *et al.*, 2013 ▸).

At around the same time, Adams, Baker and coworkers integrated the *phenix.refine* function into the popular modeling program *Rosetta* (Leaver-Fay *et al.*, 2011 ▸), which was at the time equipped with the knowledge-based force field (all-atom energy function) talaris2013 (DiMaio *et al.*, 2013 ▸). The usefulness of this method has been demonstrated for molecular-replacement models refined against low-resolution diffraction data. In recent years, this scheme has been employed to refine several dozen crystallographic structures (Birkinshaw *et al.*, 2015 ▸; Bozhanova *et al.*, 2020 ▸).

Finally, very recently the Amber ff14SB force field (Maier *et al.*, 2015 ▸) has been added as an option in *phenix.refine* (Moriarty *et al.*, 2020 ▸). This option has been tested on a set of 22 000 protein structures, leading to an appreciable improvement in a number of structure-quality metrics, but no improvement with regard to the average *R*_free_. The current implementation has limited options for molecular dynamics and no GPU acceleration. This development is too new to have led to any published applications or Protein Data Bank (PDB) records.

One may ask why state-of-the-art MD force fields have not found greater use in crystallographic refinement. In part, this is explained by the dominance of the conventional refinement tools offered by the industry-leading *Phenix* and *CCP*4 packages. However, there is also another, more fundamental reason for this situation. In protein crystals, the protein environment largely consists of an interstitial solvent: on average, solvent occupies ∼50% of the crystal volume and effectively mediates the packing of the protein molecules (Chruszcz *et al.*, 2008 ▸; Matthews, 1968 ▸). Despite this commonly known fact, all prior attempts to use MD force fields for crystallographic refinement did not include explicit or even implicit solvent.

This issue deserves an additional comment. To be precise, previous MD-based refinement protocols took into consideration a limited number of ordered water molecules found in higher-resolution structures. They also employed special models involving the so-called solvent mask to estimate the contributions from bulk solvent to structure factors (Fokine & Urzhumtsev, 2002 ▸). Nevertheless, the essential fact remains that these protocols ignored bulk solvent, *i.e.* almost all or all of the water molecules contained in the crystal, when evaluating *E*_force field_. In other words, the protein assembly has been effectively transferred into the gas phase. In this context, there are several points that are worth keeping in mind.

(i) There are no experimental methods capable of high-resolution protein structure determination in the gas phase.

(ii) MD force fields have been parameterized for use with condensed media (water) and hence are not very well suited to gas-phase simulations. Nevertheless, it has been determined that MD simulations can provide a reasonable qualitative insight into protein structures in the gas phase (Lee *et al.*, 2019 ▸).

(iii) MD data suggest that there are subtle but noticeable differences between protein structures in solution and in the gas phase. For example, many surface sites that are solvated in aqueous samples become locked in hydrogen bonds (salt bridges) in the gas phase (Patriksson *et al.*, 2007 ▸).

(iv) Therefore, it is not a very good idea to refine solvated protein structures using vacuum simulations. Indeed, it has been shown that MD-based refinement of NMR structures in explicit (or implicit) solvent produces better results than the same refinement procedure in vacuum (Xia *et al.*, 2002 ▸). One may expect the same to be true of crystallographic refinement.

Hence, in order to fulfill the potential of the advanced MD force fields one needs to include the entire body of intracrystalline water in the refinement model.

In pursuing this agenda, we have implemented a new crystallographic refinement protocol in the *Amber*16 program (Case *et al.*, 2016 ▸) using the Amber ff14SB force field. Briefly, we use the initial (unrefined) protein coordinates to construct the crystal unit cell (UC). The space between the protein molecules is filled with explicit solvent (TIP3P; Jorgensen *et al.*, 1983 ▸). The thus obtained UC is used as a simulation cell. The periodic boundary conditions used in the simulations are perfectly suited to model the periodic crystal lattice. If desired, the method can be easily extended to simulate a block of unit cells known as a supercell (Janowski *et al.*, 2016 ▸).

The simulation is controlled by the energy function (2), where *E*_force field_ represents the Amber ff14SB force field and *E*_xray_ is the maximum-likelihood (ML) target function (Afonine *et al.*, 2005 ▸; Lunin & Skovoroda, 1995 ▸), which is known for its superior properties in the context of crystallo­graphic refinement. The experimental SF data are automatically expanded to space group *P*1. Hence, the simulated UC is effectively treated as a (redefined) asymmetric unit. In this manner, structural variations can develop among all protein molecules in the simulated UC. However, deviations from the space-group symmetry remain small due to the restraining effect of the experimental SFs encoded in *E*_xray_. Generally, our model can be viewed as a realistic representation of an *individual* unit cell, rather than the traditional *ensemble-average* representation of the crystal.

There is a good reason why the data are expanded to *P*1 in our treatment. Indeed, this is the only straightforward way to include explicit interstitial solvent in MD-based refinement. To illustrate this point, consider the recently reported protocol involving the Amber ff14SB force field coupled with *phenix.refine* (Moriarty *et al.*, 2020 ▸). In this protocol, the UC is built and then evolved under the control of *E*_force field_ and *E*_xray_. However, the forces are computed only for the first asymmetric unit (ASU) and then applied to all ASUs within the UC. In this manner, the protocol maintains the original perfect symmetry of the crystal. At the same time, this scheme cannot accommodate intracrystalline water because bulk water molecules cannot be easily assigned to the individual ASUs. To circumvent this problem, in our approach we have treated the UC as a *P*1 cell.

As already indicated, the key advantage of this procedure is that it uses a highly advanced force field in conjunction with a highly realistic model of the protein crystal that is properly hydrated. The model is also well suited to represent the conformational diversity of protein molecules in the crystal lattice. Specifically, the simulated unit cell containing *N* protein molecules can be viewed as an ensemble of *N* slightly distinct conformational species. This type of representation comes on top of the standard instruments to model protein dynamics, such as *B* factors and alternate conformations, offering an attractive alternative/complement to these traditional tools. In this sense, our approach can be likened to the extensively developed ensemble-refinement methods (Burnley *et al.*, 2012 ▸; Keedy *et al.*, 2015 ▸; Levin *et al.*, 2007 ▸; Rice *et al.*, 1998 ▸), but with the advantage that in our method the conformational diversity arises ‘naturally’, *i.e.* during the MD simulation of the relevant protein crystal.

As an illustration, let us consider the interfaces between asymmetric units in the crystal lattice. In standard refinement methods (Afonine *et al.*, 2012 ▸; Murshudov *et al.*, 2011 ▸), strict symmetry is maintained between the ASUs and the interfaces between the ASUs are controlled by a simple repulsive potential that prevents steric clashes. In our approach, small dynamic variations between the ASUs are tolerated and the interfaces between the ASUs are modeled in a highly realistic fashion, complete with interstitial water. The accurate description of electrostatic and van der Waals interactions allows us to capture hydrogen bonds and salt bridges across the interfaces. The emerging picture can be rather rich in detail. For example, our model can capture the effect of cooperative conformational dynamics at the interfaces, where two side chains belonging to different protein molecules jump in a concerted fashion. All of this should help to improve the quality of the structural model, especially in areas where crystallographic electron density is poorly defined or missing.

The practical advantages of the new protocol are that it is fast (it is intended to run on GPUs) and does not involve any tunable input parameters. We have tested this protocol on a set of 84 protein structures ranging in resolution from 1.53 to 3.83 Å. The results were compared with the outcome of the extensive *Phenix*-based refinement procedure involving multiple protocols (including *Phenix* protocols employing the Amber force field). It was found that our *Amber*-based procedure consistently outperformed *Phenix* both in terms of *R*_free_ and the *MolProbity* score (Williams *et al.*, 2018 ▸). Furthermore, in ∼70% of cases the new scheme led to better *R*_free_ values than those found in the original PDB depositions. Similar favorable results were also obtained when the new scheme was tested on a set of four molecular-replacement (MR) models.

## 2. Methods

### 2.1. Refinement functionality in *Amber*

**Figure 1:**
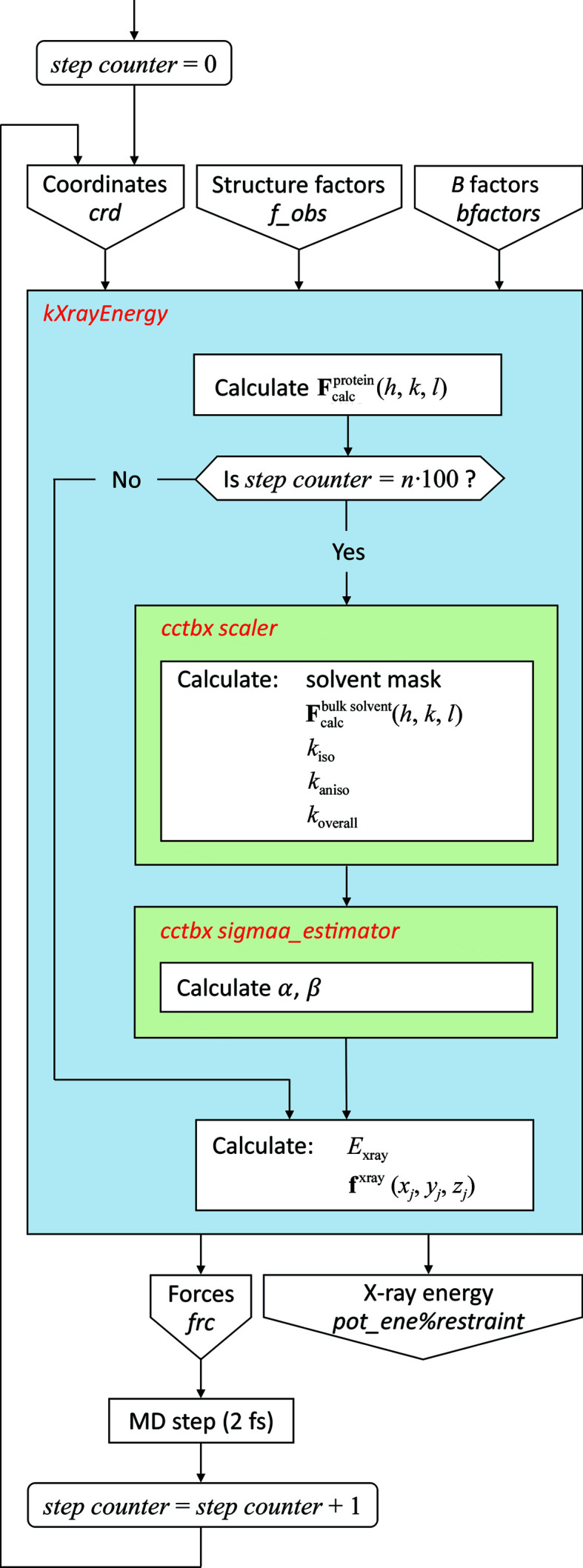
Flowchart illustrating the functionalities and interactions of the new *Amber* module *kXrayEnergy*.

A new *Amber* module has been programmed using Fortran for CPU calculations and CUDA (Kirk & Hwu, 2017 ▸) C++ code for GPU calculations. In the following, we focus on the latter version, named *kXrayEnergy*. The workflow for this module is illustrated in Fig. 1 ▸. In brief, *kXrayEnergy* receives the atomic coordinates of the structural model from the *Amber* engine. The model is the crystal unit cell, which is constructed according to the original crystal symmetry, but treated as a *P*1 cell during refinement. The cell is fully hydrated, *i.e.* the space between protein molecules is occupied by explicit water molecules. *kXrayEnergy* also reads the array with experimental structure factors *F*_obs_(*h*, *k*, *l*) and another array with atomic *B* factors (which is a part of the initial model). Additional details of the input files are provided in the next section.

The module calculates the contributions to SFs from the protein atoms, 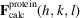 (Supplementary Equation S1). The calculations are carried out using the direct summation formula and the it1992 scattering table (Afonine *et al.*, 2012 ▸). In principle, the contributions to SFs from interstitial solvent can be calculated along the same lines because the model contains explicit solvent. However, this would require special provisions to ensure proper convergence of the results (for example averaging over multiple snapshots or using a large supercell). Instead, we opt for a simple alternative: a flat mask-based bulk-solvent model (Afonine *et al.*, 2005 ▸). *kXrayEnergy* relies on the external library *cctbx* (Grosse-Kunstleve *et al.*, 2002 ▸) to generate the solvent mask and then evaluate the contributions from bulk solvent, 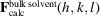 . *cctbx* is also used to calculate the scaling factors *k*_iso_, *k*_aniso_ and *k*_overall_ (Afonine *et al.*, 2013 ▸; see Supplementary Equation S2) and the ML likelihood distribution parameters α and β (Lunin & Skovoroda, 1995 ▸). Note that the flat mask-based solvent parameters *k*_iso_ and *k*_aniso_ are defined in relation to the specific resolution shells; likewise, α and β are also calculated for the individual resolution shells (but using a different binning). All of these terms are calculated once every 100 steps (0.2 ps) during the MD run; such a tactic is typical for crystallographic refinement procedures (Afonine *et al.*, 2012 ▸).

Next, *kXrayEnergy* computes the ML-based pseudo-energy *E*_xray_; because our treatment assumes space group *P*1, only the expression for acentric reflections is relevant (see Supplementary Equation S3; Afonine *et al.*, 2005 ▸). Finally, forces are computed by differentiating *w*_xray_ *E*_xray_ with respect to the coordinates of the protein atoms (Supplementary Equation S4). In doing so, we ignore the dependence of the solvent mask, 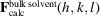 , *k*_iso_, *k*_aniso_, *k*_overall_, α and β on the protein coordinates, which is a standard approach in existing refinement schemes. The calculated forces **f** ^xray^(*x*_*j*_, *y*_*j*_, *z*_*j*_) are transmitted back to the *Amber* engine, which combines them with the force-field-based forces and uses the resultants to move the atoms. Therefore, the system is driven by both SF-based and FF-based forces, as intended [see equation (2)]. Note that **f** ^xray^(*x*_*j*_, *y*_*j*_, *z*_*j*_) are calculated according to the analytical expressions for *w*_xray_(∂*E*_xray_/∂*x*_*j*_), *w*_xray_(∂*E*_xray_/∂*y*_*j*_) and *w*_xray_(∂*E*_xray_/∂*z*_*j*_). Hence, *E*_xray_ is calculated in *kXrayEnergy* solely for the purpose of reporting. To reiterate, **f** ^xray^(*x*_*j*_, *y*_*j*_, *z*_*j*_) are applied only to the protein atoms (there is no point in applying SF-based forces to the disordered bulk solvent).

The described *kXrayEnergy* module does not have facilities to reoptimize *B* factors or to identify the ordered water molecules. Both tasks are addressed outside the core refinement protocol by using the corresponding resources from *phenix.refine* (see the next section).

Some of the functionalities of *kXrayEnergy* have already been included in the recent *Amber* release, *Amber*20, *e.g.* SF calculation using a simple version of mask-based bulk solvent (Case *et al.*, 2020 ▸). Other features are currently ported into the *Amber* distribution and will be announced later, for example the evaluation of α, β and the ML target function. All of these calculations are available in a GPU-accelerated mode.

### 2.2. Refinement pipeline

**Figure 2:**
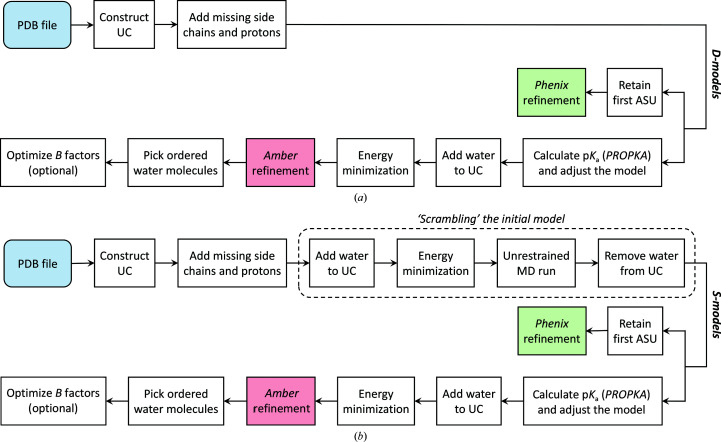
Pipeline for *Amber*-based refinement (with *Phenix*-based refinement included for the purpose of comparison). (*a*) Initial models are obtained directly from the deposited crystallographic structures (the D-set). (*b*) Initial models are from crystallographic structures subjected to a short MD run, *i.e.* intentionally ‘scrambled’ (the S-set).

The refinement pipeline is illustrated here for initial models corresponding to structures deposited in the PDB (Berman *et al.*, 2000 ▸) [the D-set; Fig. 2 ▸(*a*)] or, alternatively, for mildly ‘scrambled’ models (Rice & Brunger, 1994 ▸) [the S-set; Fig. 2 ▸(*b*)]. We begin the discussion with the former group.

The tests are conducted on crystallographic structures for which both coordinates and SF data have been deposited in the PDB. The criteria for selecting these structures are described in Section 2.4. To prepare the input files, the co­ordinates are processed using the *pdb*4*amber* utility from *AmberTools* (Case *et al.*, 2020 ▸). The values of the *B* factors are transferred from the deposited structure; the anisotropy of atomic displacement parameters (if any) is ignored. Ordered water molecules are removed. The SF data are formatted using the --write_mtz_amplitudes option of *phenix.reflection_file_converter* (Adams *et al.*, 2002 ▸). In the case of non-*P*1 structures, we expanded the deposited symmetry-reduced SF data to the *P*1 space group. This was accomplished by means of the --expand_to_p1 option. For the expanded SF data sets, we have generated new *R*_free_ flags. For this purpose we used the function --generate_r_free_flags, with 10% of all reflections assigned to the test set.

After this the chain of events is as follows.

(i) The UC is constructed using the *Amber* utility *UnitCell*.

(ii) Missing heavy atoms and hydrogens are added using the *tleap* tool. If the PDB file contains at least one atom from a given residue, all missing atoms are rebuilt; residues that are missing entirely are not rebuilt. When rebuilding a heavy atom, we assign the *B* factor of the adjacent heavy atom to it; when adding a hydrogen, we assign the *B* factor of its parent heavy atom to it.

(iii) The coordinate file is processed using *PROPKA* 3.4.0 (Olsson *et al.*, 2011 ▸) to determine the protonation state of Asp, Glu, His and Cys residues. The effective pH in the protein crystal is assumed to be the same as in the crystallization buffer (ranging from 4.0 to 9.0 for the structures in the test set). If the pH is not indicated in the PDB file, we assume a pH of 7.5 (the most frequently occurring value).

(iv) Counterions (Na^+^ or Cl^−^) and TIP3P water are added to the UC using the *AddToBox* facility. In addition to TIP3P, we also tested the more advanced TIP4P-Ew water model (Horn *et al.*, 2004 ▸), but saw no improvement in the quality of the refined structures. The number of added water molecules is determined via simple calculations using the generic protein density of 0.74 g ml^−1^ (Harpaz *et al.*, 1994 ▸). To validate this procedure, we conducted a special series of simulations on 84 test proteins using an NTP ensemble. We found that for all but two structures the volume of the simulation cell was within 2% of the target value (*i.e.* the volume of the simulated UC); on average, the volume of the simulation cell was exactly on target.

(v) Energy minimization for 500 cycles, switching from steepest descent to conjugate gradient after 50 cycles. Bonds involving H atoms are constrained using the SHAKE algorithm (Ryckaert *et al.*, 1977 ▸). The minimization is conducted using *pmemd.cuda*, with periodic boundary conditions applied to the UC and the nonbonded cutoff set to 8 Å.

(vi) *Amber*-based refinement *per se* (described in Section 2.3).

(vii) Ordered water molecules are added using the corresponding facility of *phenix.refine* (a single round with only one activated option, ordered_solvent=true). The procedure generates an *mF*_obs_ − *DF*_model_ difference density map and identifies water molecules from the relevant peaks in this map (Afonine *et al.*, 2012 ▸). As elsewhere, the it1992 scattering table and direct summation formula are used to calculate the model SFs.

(viii) Optimization of atomic *B* factors using the corresponding facility of *phenix.refine* (a single round with only one activated option, strategy=individual_adp). The procedure uses 25 steps of gradient-driven LBFGS minimization.

The last step, (viii), is optional in the following sense. If this step leads to an improvement in *R*_free_, then its output is taken to be the final product of the pipeline shown in Fig. 2 ▸(*a*). Conversely, if step (viii) does not improve *R*_free_ then this step is disregarded and instead the output of step (vii) is treated as the final model. Note that strictly speaking *R*_free_ should not be used as a guide to select the best structure. In practice, however, this does not compromise the integrity of the process (Urzhumtsev & Lunin, 2019 ▸) and is widely used in various refinement algorithms, including those in *Phenix*. Note also that from a structural biology standpoint improvement in *R*_free_ does not directly correlate with success of the refinement. For example, the refinement of a key catalytic residue in the active site of the enzyme is of great value, even though the associated improvement in *R*_free_ may be minimal.

In addition to models extracted directly from the PDB, we also use intentionally distorted models (the S-set). The pipeline shown in Fig. 2 ▸(*b*) includes a series of steps whereby such ‘scrambled’ models are manufactured (dashed box in the plot). Briefly, the hydrated UC is prepared in the same manner as described above. It is then heated from 0 to 298 K over 20 ps with 10.0 kcal mol^−1^ Å^−2^ harmonic restraints applied to all protein atoms. After this, the system is evolved for 100 ps using unrestrained molecular dynamics. The MD simulation parameters are the same as described in the next section (but with *w*_xray_ = 0). The output from this step is the final MD frame, which deviates from the original crystallographic structure and thus imitates an imperfect initial model in need of refinement (Rice & Brunger, 1994 ▸). We also used two variations of this scheme in which the length of the MD trajectory was increased to 1 ns or to 10 ns. The resulting scrambled models were termed S1 and S2, respectively. The refinement of the S-models [Fig. 2 ▸(*b*)] follows exactly the same scheme as described above for the D-models [Fig. 2 ▸(*a*)].

It should be noted that the step involving the calculation of p*K*_a_ (see Fig. 2 ▸) is only marginally useful. Indeed, this type of calculation has rather limited accuracy. Firstly, we usually know the pH of the crystallization buffer, but not the pH of the interstitial solvent in the crystal, which is relevant to the problem at hand. Secondly, structure-based programs such as *PROPKA* produce substantial errors at the level of ±1 pH unit (Davies *et al.*, 2006 ▸). Thirdly, such calculations are best performed in the context of a large supercell (Kurauskas *et al.*, 2017 ▸). One may expect that this particular aspect of MD-based refinement can be improved in the future.

### 2.3. Refinement protocol

**Figure 3:**
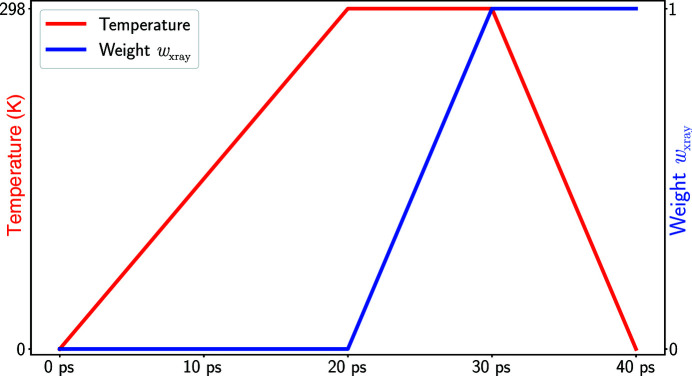
A schematic diagram of the *Amber*-based refinement protocol used in this work. This particular schedule has been developed through extensive experimentation and appears to be near-optimal for the goals of this study.

In this section, we describe the refinement step represented by the pink box in Fig. 2 ▸. The refinement involves a short MD run employing SF-based restraints [equation (2)]. The modified *Amber* program is used as described in Section 2.1. The scheme of the refinement protocol is presented in Fig. 3 ▸, where the red line represents temperature (with the scale given on the left) and the blue line represents *w*_xray_ (with the scale given on the right). The procedure begins with a 20 ps heating period whereby the temperature is raised from 0 to 298 K. During this period all protein atoms are restrained with 10.0 kcal mol^−1^ Å^−2^ harmonic restraints. SF restraints are not employed, *w*_xray_ = 0. During the next 10 ps SF restraints are gradually introduced into the system, with *w*_xray_ ramped up from 0.0 to 1.0. The value *w*_xray_ = 1.0 has been recommended for crystallographic refinement based on fundamental thermodynamics considerations (Fenn & Schnieders, 2011 ▸). We tested other settings, 0.5 or 2.0, and determined that the plateau value of 1.0 produces the best results in the context of this particular protocol. The final stage is cooling, whereby the temperature is gradually lowered from 298 to 0 K while maintaining full-strength SF restraints with *w*_xray_ = 1.0. The purpose of this step is to get rid of local dynamic fluctuations, while steering the system into the global energy minimum corresponding to the refined structure (taken to be the final MD frame).

As indicated above, the SF restraints in this procedure are derived from the ML-based pseudo-energy (Supplementary Equation S3). The (resolution-shell-dependent) ML distribution parameters α and β are updated every 100 steps (0.2 ps) during the MD run. Similarly, the mask used to calculate the bulk-solvent contribution to SFs is also updated every 100 steps (0.2 ps).

The simulation is conducted using the NVT ensemble, with periodic boundary conditions applied to the unit cell (taken to be the simulation cell). Bonds involving hydrogens are restrained by means of the SHAKE algorithm. The non­bonded cutoff is 8 Å (a value of 10.5 Å was also tested). Long-range electrostatic interactions are treated using the particle mesh Ewald summation scheme with default parameters for grid spacing and spline interpolation. The temperature is controlled by means of the Langevin thermostat (Izaguirre *et al.*, 2001 ▸) with collision frequency γ = 2 ps^−1^. The simulations were conducted using in-house GPU workstations under CUDA MPS.

### 2.4. Protein test set

A set of 84 crystallographic protein structures, ranging in resolution from 1.53 to 3.83 Å, were used to test and validate the new refinement procedure (see Supplementary Table S1 for a complete list of the structures and their basic statistics). This set resulted from a comprehensive search of the PDB subject to the following criteria.

(i) The crystal structure contains only protein chains without modified residues or nonprotein ligands. This restriction stems from the lack of force-field parameters for many diverse protein modifications and ligand molecules.

(ii) The protein chains should be free of gaps (*i.e.* at least one heavy atom per residue should be resolved). Although it is possible to rebuild the missing fragments, we leave this option for future investigation.

(iii) Experimental diffraction data have been deposited (either in the form of SFs or scattering intensities).

(iv) There is no crystal twinning. In the future, this restriction can be removed by redefining the ML target function and its derivatives.

(v) The atomic occupancies are all equal to 1.0. In principle, protein molecules sampling different alternate conformations can be used to populate the unit cell, but this will further complicate the protocol.

(vi) The protein mass per ASU is less than 40 kDa and the UC volume is less than 200 000 Å^3^. We seek to construct a compact set of structural models where all computations will take no longer than several days. Otherwise, if the goal is to refine an individual structure, this requirement can be ignored.

(vii) All unit-cell dimensions are longer than a doubled non­bonded cutoff distance, *i.e.* 16 Å. Modeling smaller crystal cells using a GPU can cause complications (Case *et al.*, 2020 ▸).

(viii) The number of ordered water molecules in the structure (ASU) is less than 50. Our core refinement procedure does not involve any ordered waters (which are rebuilt at a later stage; see Fig. 2 ▸). We seek to limit potential biases due to the removal of the ordered water molecules by limiting the number of these molecules. Plans to improve the modeling of crystallographic water are outlined in Section 5.

As can be appreciated from the above comments, none of the restrictions (i)–(viii) are of a fundamental nature. For instance, force-field parameters for typical precipitants and ions occurring in the crystallographic structure can either be found in the literature or determined using a number of well established tools (Wang *et al.*, 2004 ▸, 2006 ▸).

The set of 84 protein structures obtained via the selection procedure described above has further been used in two different forms: as is (the D-set) or in a mildly distorted form following a short unrestrained MD run (the S-set; see Section 2.2 for details). To characterize the quality of the S-models, we calculated their atomic r.m.s.d.s relative to the original crystallographic structures (including all protein chains; limited to those atoms that are found in the original PDB file). The average all-heavy-atom r.m.s.d. for proteins in the S-set proved to be 1.06 Å. The more distorted S1-set and S2-set feature average r.m.s.d.s of 1.32 and 1.55 Å, respectively. For reference, it is generally accepted that an r.m.s.d. of 1.5 Å between the search model and the target is the limit for the MR method to be usable (Scapin, 2013 ▸).

*R* factors reported in this work were calculated using the corresponding *MolProbity* function, as available in *Phenix*. However, when comparing the results of our refinement procedure with the original PDB depositions, we used the value of *R*_free_ as indicated in the PDB records. For ten structures these values could not be found in the PDB records and therefore the comparison was limited to the remaining 74 structures.

### 2.5. Comparison with *Phenix* refinement

To assess the results of our *Amber*-based refinement procedure, we compared them with the results obtained by *Phenix*, which is one of the two leading software platforms for protein crystallography. *Phenix* offers many different options for structure refinement. In the interests of a fair comparison, we sought to test as many of these options as practicable. Specifically, for each PDB model we conducted 32 *Phenix* runs using different refinement schemes and subsequently selected the one that produced the lowest *R*_free_ for comparison with *Amber*. The following options in *Phenix* have been systematically tested.

(i) *Phenix* equipped with the CDL force field versus the Amber ff14SB force field.

(ii) Simulated-annealing (SA) scheme: SA using torsional angle coordinates versus SA using Cartesian coordinates versus SA using torsional angle coordinates followed by SA using Cartesian coordinates versus no SA.

(iii) *w*_xray_ optimization (WO) during the course of refinement versus no *w*_xray_ optimization.

(iv) *B*-factor optimization (BO) during the course of refinement versus no *B*-factor optimization.

Note that *Phenix* protocols have certain special features. For example, the WO+BO combination invokes the specialized target function *w*_xray_ *E*_xray_ + *E*_bf_ (Afonine *et al.*, 2012 ▸), where *E*_bf_ represents the set of empirical restraints imposed on *B* factors and *w*_xray_ is treated as an optimizable parameter.

In the 32 *Phenix* runs per structure, we implemented all possible combinations of the above selections. The success rates of the different protocols are shown in Supplementary Table S2.

The calculations were conducted using *Phenix* 1.18.2-3874. Each individual *Phenix* run consisted of five macrocycles. This setting produced the best overall results in our preliminary trials, where we tested values of between three (the default) and seven. Other parameters, as indicated below, were set to *Phenix* default settings. The same (highly efficient) ML target function was used as in our *Amber*-based approach. The ML parameters α and β were updated at the beginning of each macrocycle. Likewise, the solvent mask was updated at the beginning of each macrocycle; the algorithm to compute the bulk-solvent contribution was the same as that invoked in our *Amber*-based protocol. Each macrocycle involved 25 rounds of gradient-driven LBFGS coordinate minimization and the real-space refinement step (for details, see Afonine *et al.*, 2012 ▸). Following the default *Phenix* arrangement, the second and and penultimate (*i.e.* fourth) macrocycles additionally include SA treatment (for those schemes where SA was selected; see above). For the protocols that use the BO option, each macrocycle ends with the optimization of isotropic *B* factors.

All *Phenix* calculations used the direct summation option to compute SFs and the it1992 scattering table. These are high-accuracy options analogous to those used in our *Amber*-based protocol. Riding H atoms are used throughout the calculations and the automatic correction of flipped Asn/Gln/His side chains has been applied. Ordered water molecules are added to the model after all refinement macrocycles are completed (alternatively, it is possible to refine coordinate sets containing ordered water, but in our limited tests we found no advantage in doing this). There is only one major refinement option that is available in *Phenix* but has not been included in the current procedure: TLS modeling. This was a deliberate choice since TLS refinement typically leads to formal solutions that are inconsistent with the physical assumptions of the TLS model (Urzhumtsev & Lunin, 2019 ▸).

Since different PDB structures use a different fraction of data to compute *R*_free_ and some of the structures do not report *R*_free_ flags, we chose to regenerate these flags for all of the structures at hand. For this purpose we used the *Phenix* function --generate_r_free_flags, with 10% of all reflections assigned to the test set, as in the *Amber*-based procedure (see Section 2.2).

Considering the refinement of all models in the S-set and the D-set, the *Phenix*-based calculations in this study involved 5376 individual *Phenix* runs. A small fraction of these runs failed to complete properly. Specifically, three models ran into trouble because of the atoms on special positions. This complication was successfully handled by *Phenix* using the CDL force field, but not by *Phenix* using the Amber ff14SB force field. Aside from this, 24 runs also aborted for various reasons. Altogether, only 1.3% of the individual *Phenix* runs resulted in abnormal termination, which is unlikely to have any material impact on the outcome of the analyses.

### 2.6. Refinement of molecular-replacement models

For several proteins in our data set, we constructed MR models using the same coordinates as were used to build the MR models in the original structural studies. Specifically, we selected the following (target, model) pairs: (5xbh, 2pbr), (5arj, 5ar6) and (4c0m, 4bsx). The sequence-identity levels in these pairs were 99.5%, 97.5% and 96.8%, respectively. In the case of PDB entry 5arj, the construct contained an N-terminal histidine tag, which is unresolved in the crystal structure and absent from PDB entry 5ar6. To pre-process the MR models, we took the following steps.

(i) We treated the initial model using *phenix.sculptor* (Bunkóczi & Read, 2011 ▸). The program matches chain *A* of the model with the FASTA sequence of the target (deleting terminal residues, if necessary) and conducts point mutations according to the sequence, *i.e.* it renames mutated residues and deconstructs their side chains.

(ii) We treated the obtained model with *phenix.phaser* (McCoy *et al.*, 2007 ▸) to assemble the desired ASU. At this step the sequence identity between the target and *phenix.sculptor*-edited models is 100%. We used the ‘full search’ mode for this manipulation.

(iii) We applied *phenix.autobuild* (Terwilliger *et al.*, 2008 ▸) to rebuild the mutated side chains and optimize the coordinates as directed by the electron-density map. We used the setting rebuild_in_place=True, with the corresponding default options replace_existing=True and include_input_model=True. We also requested one round of *B*-factor refinement: refine_b=True and strategy=individual_adp. All other refinement options were disabled. The resulting models were reasonably close to the target structures, with heavy-atom r.m.s.d.s of 1.16, 0.63 and 1.28 Å for the (5xbh, 2pbr), (4c0m, 4bsx) and (5arj, 5ar6) pairs, respectively.

In addition, we also considered a pair (4ug3, 4ug1) where the sequence identity is significantly lower at 57.9%. We treated this pair using the same protocol as described above, except for the *autobuild* stage, where we allowed a full-scale refinement. Specifically, the default set of refinement flags was employed, including refine_xyz=True, refine_final_model_vs_orig_data=True *etc*. The resulting model showed a heavy-atom r.m.s.d. of 2.15 Å from the target structure.

The thus obtained MR models were subsequently refined by means of the *Amber*-based procedure, as described in Sections 2.2 and 2.3. For the sake of comparison, they were also refined using the *Phenix* scheme (Section 2.5).

## 3. Results

### 3.1. Example of *Amber*-based refinement

To illustrate the performance of the *Amber*-based refinement routine, we chose the structure of the complex between ubiquitin and ubiquitin-conjugating enzyme E2-25K (PDB entry 3k9p; Ko *et al.*, 2010 ▸), which belongs to our test set of 84 protein structures. The structure is monoclinic (space group *P*12_1_1), with the ASU containing a single copy of the complex and the UC containing two such copies. The resolution is reported as 2.80 Å, with *R*_work_ = 0.232 and *R*_free_ = 0.296. The structure contains no crystallographic water.

To test the different refinement procedures, we used the automatically generated S-model characterized by a heavy-atom r.m.s.d. of 1.16 Å and a C^α^ r.m.s.d. of 0.80 Å relative to the original PDB entry 3k9p coordinates. The *R*_work_ and *R*_free_ values for this model are both 0.42 (for the scrambled model, the distinction between *R* and free *R* is lost).

**Figure 4:**
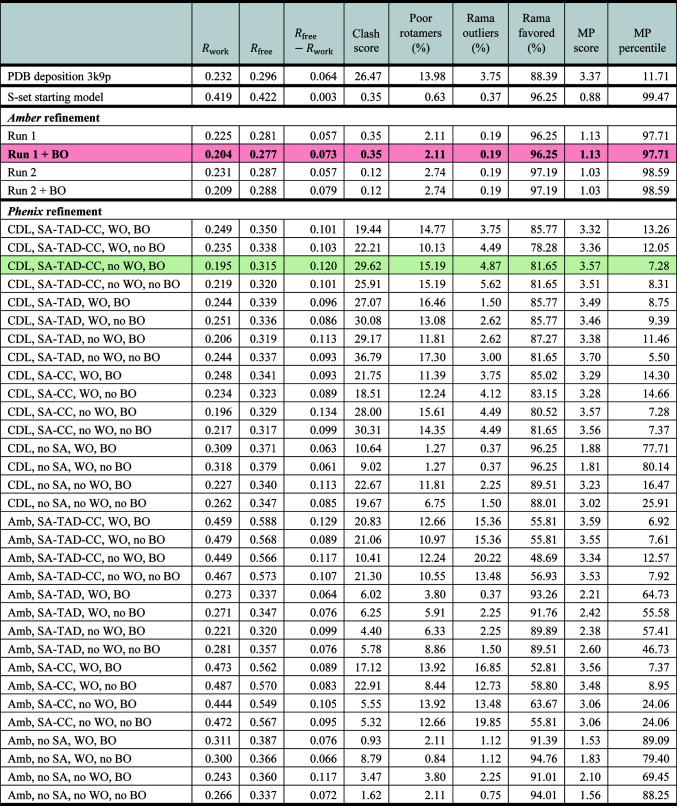
Structural statistics of the crystallographic structure with PDB code 3k9p, the derivative scrambled model, four models from the *Amber*-based refinement procedure and 32 models from the *Phenix*-based refinement procedure. Abbreviations: Rama, Ramachandran; MP, *MolProbity*; CDL, conformation-dependent library; Amb, Amber ff14SB; SA, simulated annealing; TAD, torsional angle dynamics; CC, Cartesian coordinates; WO, weight optimization; BO, *B*-factor optimization (see Section 2.5 for further details). The best (*i.e.* lowest *R*_free_) *Amber*-refined model is indicated by pink shading and the best *Phenix*-refined model by green shading.

In Fig. 4 ▸ we summarize the outcomes of the refinement procedures. In brief, we perform two *Amber*-based refinement runs (which differ in randomly generated initial velocity distributions). Each run is followed by a round of *B*-factor optimization. This results in four possible choices (two models before the *B*-factor optimization step and two models after the *B*-factor optimization step). From these four possibilities we select the one with the lowest *R*_free_, which is taken to be the final product of the *Amber*-based refinement procedure (the pink shaded row in Fig. 4 ▸). The *R*_free_ value of this MD-refined model, 0.277, is an appreciable improvement over the value that is reported in the PDB deposition, 0.296. Furthermore, the MD-refined model is also improved in terms of generalized structure-quality metrics, such as clashscore, the number of residues in the most/least favored regions of the Ramachandran map *etc*. These parameters are conveniently combined into a single measure: the *MolProbity* score. For the refined model, this score is 1.13, corresponding to the 98th percentile for structures of comparable resolution. This is in contrast to the PDB-deposited structure, which has a score of 3.37, which translates into the 12th percentile. The favorable *MolProbity* scores are as expected for structures refined using the MD platform, providing a nice bonus to the lowered *R*_free_.

As a next step, we sought to compare the result of the *Amber*-based refinement with that of the industry-standard *Phenix*-based refinement. As described in Section 2.5, we have implemented 32 different *Phenix* protocols systematically testing different combinations of input parameters. These parameters pertain to the restraints used (*i.e.* the force field), the simulated-annealing schedule, the handling of *w*_xray_ and the handling of *B* factors. Of the 32 obtained models, we selected the one with the lowest *R*_free_ (the green shaded row in Fig. 4 ▸). This model is considered to be the product of the *Phenix* refinement procedure. The *R*_free_ value for this model, 0.315, falls short of that of the deposited model, 0.296, and that of the *Amber*-refined model, 0.277. The *MolProbity* score, 3.57, is also dissatisfying, corresponding to the 7th percentile. Therefore, for this particular example *Amber*-based refinement performs better than *Phenix*-based refinement.

A closer look at the *Phenix*-refined model also finds a substantial difference between *R*_free_ and *R*_work_ of 0.120, which is suggestive of overfitting. To address this problem, we additionally carried out a series of refinement runs consisting of three macrocycles, but found no improvement (not shown). An overview of the 32 *Phenix* protocols (see Fig. 4 ▸) reveals a group of failed protocols with *R*_free_ values in the range 0.55–0.59. All of these protocols employ the Amber ff14SB force field in conjunction with an SA scheme involving Cartesian coordinates (SA-CC or SA-TAD-CC). We also observed this behavior for other crystallographic structures; this is likely to be a technical issue with the recent incorporation of the Amber force field into *Phenix*.

At this point, it is timely to compare the speed of the *Amber*- and *Phenix*-based computations. To benchmark the speed, we used a workstation equipped with eight NVIDIA GeForce RTX 2080 Ti graphics cards, two Intel Xeon Silver 4210 CPUs (2 × 10 cores at 2.2 GHz) and 128 GB RAM. Using two GPU cards, all of the *Amber*-based computations listed in Fig. 4 ▸ took a total of 1 h 42 min. In contrast, using two CPUs (running the total of 40 threads and simultaneously executing 40 refinement protocols), all of the *Phenix-*based computations listed in Fig. 4 ▸ took a total of 12 h 22 min. Therefore, for this particular setting the *Amber*-based refinement is not only preferable in terms of key quality metrics, but is also nearly an order of magnitude faster.

This last statement, however, should be qualified. Firstly, *Phenix*-based refinement can be executed on virtually any computer, while *Amber*-based refinement requires a GPU workstation (the same refinement procedure implemented on a CPU is roughly two orders of magnitude slower). While GPU workstations have become rather commonplace in research laboratories, they are still not universally available. In part, this problem can be addressed by setting up a GPU-enabled web server dedicated to *Amber*-based refinement jobs (as discussed in Section 5). Secondly, the *Phenix* procedure can be downsized from 32 protocols to a smaller number of protocols. As already mentioned, eight *Phenix* protocols involving SA-CC or SA-TAD-CC schedules under Amber ff14SB can be excluded. To save time, one can also omit some of the less productive *Phenix* protocols. For instance, in all of our tests on the S-set and D-set models the {protocol Amb, SA-TAD, WO, no BO} was only once the winning protocol (see Supplementary Table S2). However, by eliminating such protocols one may potentially miss the best solution (*i.e.* there is a trade-off between computation time and the quality of the refined model).

Finally, this example can be used to discuss one of the important characteristics of the system: the data-to-parameter ratio. For the original structure with PDB code 3k9p this ratio amounts to 0.8. In the *Amber*-based protocol, the entire UC, which consists of two ASUs, is treated as a new asymmetric unit. Accordingly, the number of structural variables and independently adjustable *B* factors is doubled. At the same time, expanding the data from *P*12_1_1 to *P*1 doubles the size of the SF data set. In other words, those SF-based restraints that were previously fulfilled automatically due to the space-group symmetry now become fully relevant. Therefore, formally speaking, the data-to-parameter ratio in the new protocol remains unchanged compared with its conventional counterpart. Thus, the relative success of the new refinement scheme cannot easily be explained away by the increased number of adjustable parameters.

### 3.2. Summary of *Amber*-based refinement tests

**Figure 5:**
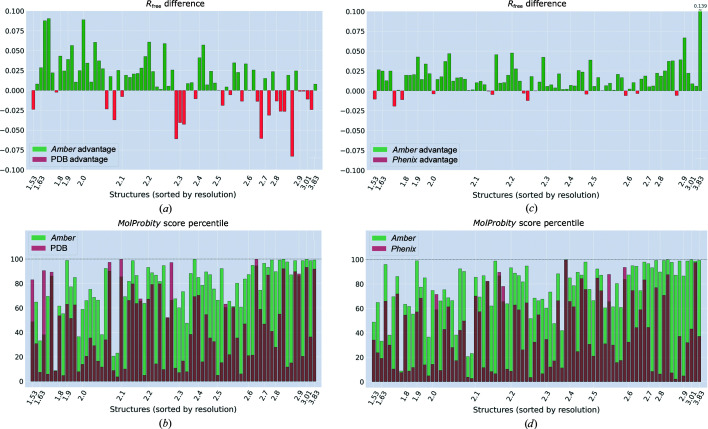
Summary of the refinement results starting from the scrambled models. All data shown in these graphs are also tabulated in Supplementary Table S3. (*a*) Difference in *R*_free_ values between the original PDB depositions and the structures obtained through *Amber*-based refinement of the S-models. The data are from 74 test-set structures where *R*_free_ is reported as part of the PDB deposition (sorted in this plot according to the crystallographic resolution). A green color indicates that the *Amber*-refined structure is superior to the original PDB structure and a red color indicates that the *Amber*-refined structure is inferior to the original PDB structure. (*b*) *MolProbity* score percentiles for the structures obtained through *Amber*-based refinement of the S-models (semi-transparent green bars) and the original PDB structures (semi-transparent red bars). Of note, the *MolProbity* scores of the *Amber*-refined structures are somewhat adversely affected by the addition of ordered water (see Fig. 2 ▸): before this step the average *MolProbity* score percentile is 86th, while after this step it drops to 76th. (*c*) Difference in *R*_free_ values between the structures obtained through *Phenix*- and *Amber*-based refinement of the S-models. The data are from 84 test-set PDB entries. An exceedingly high Δ*R*_free_ of 0.139 (rightmost bar in the plot) reflects the failure of *Phenix* for this particular structure. (*d*) *MolProbity* score percentiles for the structures obtained through *Ambe*r- and *Phenix*-based refinement of the S-models.

The same procedure as described above for PDB entry 3k9p was performed for the other protein structures in the test set (see Section 2.4). The obtained results are summarized in Fig. 5 ▸. Fig. 5 ▸(*a*) compares the *R*_free_ values obtained as a result of *Amber*-based refinement of the scrambled models (S-models) with the *R*_free_ values reported in the original PDB depositions. The green bars in the plot indicate an advantage of the *Amber*-refined structures, while the red bars indicate an advantage of the original PDB coordinates. Quite remarkably, the *Amber*-based procedure, which begins with rather poor initial models (heavy-atom r.m.s.d. of 1.06 Å), in most cases achieves a significant improvement over the PDB-deposited structures. The average improvement, Δ*R*_free_, amounts to 0.012. Fig. 5 ▸(*b*) contains information on the *MolProbity* score, which is viewed as a secondary parameter of interest. As can be seen from the plot, *Amber*-refined structures are typically better regularized compared with the original PDB structures (cf. the green and red bars). On average, *Amber*-refined structures are in the 76th percentile, while the original PDB structures are in the 44th percentile for the *MolProbity* score [calculated for the structures in Figs. 5 ▸(*a*) and 5 ▸(*b*)]. Altogether, these results demonstrate the success of the *Amber* refinement scheme.

The data in Figs. 5 ▸(*a*) and 5 ▸(*b*) also reveal a certain general trend. Specifically, for higher-resolution structures (the left side of the plot) the *Amber* procedure achieves a significant improvement in *R*_free_, but produces only moderately good *MolProbity* scores. In contrast, for lower-resolution structures (the right side of the plot) our procedure fails to significantly improve *R*_free_, but achieves near-perfect *MolProbity* scores. This can be understood by considering that the diffraction data for high-resolution structures contain a greater number of SFs compared with low-resolution structures. Consequently, high-resolution structures are shaped by *E*_xray_ to a greater extent, at the expense of *E*_force field_, while for low-resolution structures the balance is tilted in the opposite direction. This observation leads us to suggest that the outcome of the *Amber*-based refinement procedure can be further improved by making *w*_xray_ resolution-dependent. Specifically, we envisage that *w*_xray_ can be increased for low-resolution data sets. We will defer such experimentation to future work.

A closer look at the data offers a more nuanced perspective on the results of Figs. 5 ▸(*a*) and 5 ▸(*b*). Consider, for example, the structure with the largest *R*_free_ improvement, PDB entry 1ae2 (Su *et al.*, 1997 ▸; originally reported *R*_free_ of 0.326, after refinement 0.236). This structure can be readily improved not only by our *Amber*-based procedure, but also by *Phenix* and *PDB-REDO* (Joosten *et al.*, 2009 ▸, 2014 ▸). There are a number of other PDB structures in our test set that afford similar easy improvements. On the other hand, consider the case where *R*_free_ experiences the most significant deterioration, PDB entry 2jee (Ebersbach *et al.*, 2008 ▸; originally reported *R*_free_ of 0.321, after refinement 0.404). For this structure, the reported *R*_free_ value is very close to *R*_work_, 0.321 versus 0.310, which suggests a technical error in the original *R*_free_ determination (Wang, 2015 ▸). Hence, in this particular case the performance of our *Amber*-based procedure is likely to be somewhat better than it appears from the plot. There are also several other structures in the PDB test set which seem to suffer from the same problem, *i.e.* the reported *R*_free_ values are lower than can be reasonably expected.

Note that in this comparison, Figs. 5 ▸(*a*) and 5 ▸(*b*), the bar is set sufficiently high. Indeed, we start with a rather poor S-model and apply essentially a single standardized *Amber*-based protocol to this model. We then compare the results with the *bona fide* PDB structures, which are usually refined with much care (including certain tools which we do not use, such as the TLS scheme). The situation is further complicated by misestimated *R*_free_ values, which are occasionally found in the PDB structures (see above). This prompts us to turn to a different type of comparison. Namely, we compare the results of *Amber*- and *Phenix*-based refinement procedures. The results are summarized in Figs. 5 ▸(*c*) and 5 ▸(*d*) using the same format as before. As can be seen from Fig. 5 ▸(*c*), the *Amber*-based refinement procedure is almost always preferable to its *Phenix*-based counterpart. The average improvement in the free *R* factor, Δ*R*_free_, amounts to 0.016. The *MolProbity* metric also favors *Amber*-refined structures over *Phenix*-refined structures, with an average *MolProbity* score percentile of 76th versus 41st. Finally, bear in mind that *Amber* calculations are much faster, roughly by a factor of five. This is a significant advantage, given that the *Phenix* calculations to generate Fig. 5 ▸ required two weeks of time using 40 Intel CPU cores. Based on this evidence, we conclude that our *Amber*-based refinement procedure compares favorably with the similar *Phenix* scheme.

**Figure 6:**
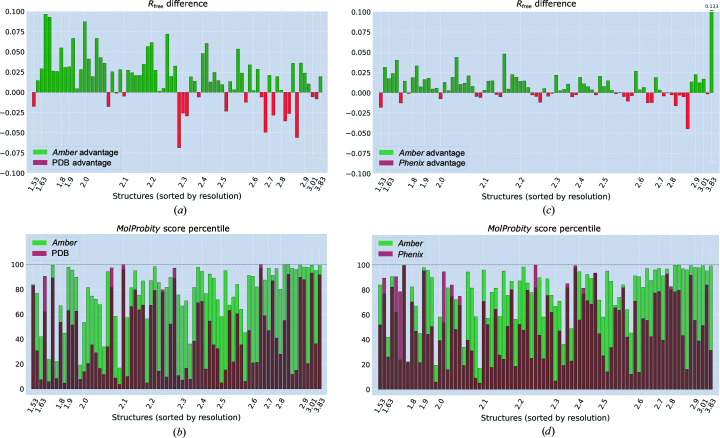
Summary of the refinement results starting from the deposited models (D-models). All data shown in these graphs are also tabulated in Supplementary Table S3. Plotting conventions are the same as in Fig. 5 ▸.

In the above tests we have used scrambled models, as initially proposed by Rice & Brunger (1994 ▸). It should be pointed out, however, that in the context of our study the use of S-models can in principle be questioned. Indeed, one may speculate that structures that have been distorted via a short MD run can subsequently be remedied using another MD run. To elaborate, each S-model represents an MD snapshot reflecting a multitude of various harmonic fluctuations (which occur during the scrambling trajectory). Our refinement protocol involves the cooling stage (see Fig. 3 ▸), whereby the fluctuations are dissipated and the system evolves towards the minimum-energy structure. This invites the question: is it fair to use *Amber*-generated S-models to test our *Amber*-based refinement procedure? To address this question, we repeated the above tests using the deposited PDB coordinates as our initial models (D-models). Clearly, these coordinates have nothing to do with the *Amber* software and, therefore, such tests should be in no way biased toward the *Amber*-based refinement routine. The results of the tests using D-models are summarized in Fig. 6 ▸.

An inspection of the data in Fig. 6 ▸(*a*) indicates that *Amber* improves the *R*_free_ value for 56 out of 74 PDB structures. The average improvement, Δ*R*_free_, amounts to 0.018. This is a considerable decrease in *R*_free_, signifying the success of the *Amber*-based procedure. The result is noticeably better than that obtained previously for the S-models, where Δ*R*_free_ is 0.012. This is to be expected given that the S-models are of rather poor quality, which makes them harder to refine.

Similar conclusions can be drawn from inspection of the *MolProbity* indicators [Fig. 6 ▸(*b*)]. The average *MolProbity* score percentile before and after refinement is 44th versus 78th. This is a substantial increase in this generalized measure, which characterizes the ‘goodness’ of the protein structure. At the same time, this is only slightly improved compared with Fig. 5 ▸(*b*), where the same quantities are 44th versus 76th. In this sense, our *Amber*-based procedure performs equally well for high- and low-quality initial models.

Another type of comparison, between *Amber*- and *Phenix*-based refinements, is illustrated in Figs. 6 ▸(*c*) and 6 ▸(*d*). As can be seen from Fig. 6 ▸(*c*), *Amber* still consistently outperforms *Phenix*. However, the average Δ*R*_free_ is only 0.008, much less than the value obtained previously for the S-models, 0.016. In other words, our *Amber*-based procedure is only moderately more successful than *Phenix* when applied to the high-quality D-models, but is substantially more successful when applied to the lower-quality S-models. Clearly, it is the ability to deal with imperfect models that is of primary importance for refinement software. Finally, if we turn to the *MolProbity* scores, Fig. 6 ▸(*d*), we find the same trend there. Our *Amber*-based refinement procedure outscores the *Phenix* scheme, with an average *MolProbity* score percentile of 78th versus 53rd. For comparison, refinement of the S-models produced values of 76th versus 41st. Once again we observe that *Amber* holds a major advantage over *Phenix* when applied to poor initial models, but the gap narrows when dealing with more accurate models.

### 3.3. *Amber*-based refinement of MR models

As an example of a more practical application, we performed *Amber*-based refinement on a number of MR models (see Section 2.6). For instance, our test set includes the structure of a mutant hyperthermophilic thymidylate kinase, PDB entry 5xbh (Biswas *et al.*, 2017 ▸). It was originally solved using the corresponding wild-type structure, PDB entry 2pbr, as a molecular-replacement model. In the following, we seek to replicate the original structure-solution process. We begin by preparing the MR model. For this purpose, the coordinates of PDB entry 2pbr have been treated with *phenix.sculptor*, then with *phenix.phaser* and finally with *phenix.autobuild* (see Section 2.6). The resulting MR model is reasonably accurate, with a heavy-atom r.m.s.d. to the target structure of 1.16 Å. We refined this model using the same *Amber*-based procedure as employed above. For the sake of comparison, we also refined it using the same *Phenix* scheme as above. In both cases, the refinement is driven by the experimental SF data from PDB entry 5xbh (the target).

The two refinement procedures converged to near-identical solutions, with an *R*_free_ of 0.265 and 0.266, respectively. Both came ahead of the PDB-deposited structure 5xbh, which had an *R*_free_ of 0.281. In this particular case, the *Amber*-refined structure has a mediocre *MolProbity* score, corresponding to the 52nd percentile, falling behind both the *Phenix*-refined structure and the PDB-deposited structure.

Our next case study involved the (target, model) pair (4c0m, 4bsx) representing the N-terminal domain of the protein TRIF (Ullah *et al.*, 2013 ▸). The difference between the two constructs is three single-residue substitutions. The obtained MR model in this case is of very high quality, with a heavy-atom r.m.s.d. to the target of only 0.63 Å. Of note, *Amber* was distinctly more successful than *Phenix* in refining this model, achieving an *R*_free_ of 0.303 against 0.329 for *Phenix*. In fact, *Amber* produced a considerably better result than it achieved previously with S-models and D-models (0.317 and 0.318, respectively). While the *Amber* result falls short of the originally reported *R*_free_ value, 0.291, it compares favorably with the updated *R*_free_, which is also listed in the PDB record, 0.309. The *MolProbity* score of the *Amber*-refined structure was near-perfect, in the 100th percentile, with the *Phenix*-refined and PDB-deposited structures not far behind.

Yet another example is the pair (5arj, 5ar6) representing porcine RNase 4 (Liang & Acharya, 2016 ▸). The two constructs differ by three amino acids; aside from this, the target contains a 12-residue N-terminal tag which is unresolved in the structure and absent from the model construct. The obtained MR model in this case is moderately accurate, with a heavy-atom r.m.s.d. from the target of 1.28 Å. For this pair, our *Amber*-based refinement scheme is clearly superior to its *Phenix* counterpart, achieving an *R*_free_ of 0.289 versus 0.333. Furthermore, the accuracy of the *Amber*-refined structure is comparable to that of the PDB-deposited structure, which has an *R*_free_ of 0.282. As is usually the case, the *Amber*-refined structure also has the best *MolProbity* score, in the 92nd percentile.

The above three examples make use of initial models that are near-identical to the targets, with a sequence identity of 97% or higher. Now we address a more challenging case: (4ug3, 4ug1) (Rismondo *et al.*, 2016 ▸). These structures represent the same protein, the N-terminal domain of the cell division regulator GpsB, but from two different bacteria. The sequence identity of the two proteins is 58%; there is also a two-residue deletion in PDB entry 4ug3 (residues 11–12), which requires some additional rebuilding of side chains when working on an MR model. For this system we have used a more aggressive model-building strategy (see Section 2.6), resulting in an MR model that is 2.15 Å away from the target. This relatively poor level of agreement represents a considerable challenge for standard refinement algorithms (Urzhumtsev & Lunin, 2019 ▸).

In this more demanding situation, *Amber* again outperforms *Phenix*, with an *R*_free_ of 0.305 versus 0.333. Furthermore, the accuracy of the *Amber*-refined structure approaches that of the PDB-deposited structure 4ug3, which has an *R*_free_ of 0.301. While this is a satisfactory result, there is room for further improvement. We noticed that the problematic area of the MR model (inherited by the *Amber*-refined structure) is the termini. Since the initial model was constructed using the *rebuild_in_place* option with chain *A* from PDB entry 4ug1 as a template, all four of its protein chains are comprised of residues −2 to 65. Incidentally, a number of terminal residues in this model lack adequate electron density. Guided by per-residue map correlation coefficients, we deleted such terminal residues from the MR model. The resulting trimmed model was subjected to *Amber* refinement. This time the refinement led to an *R*_free_ of 0.291, which is considerably better than the value reported in the PDB-deposited structure. The final *Amber*-refined structure also boasts a perfect *MolProbity* score corresponding to the 100th percentile.

Generally, the type of problem that is described in this section is difficult to deal with for any refinement routine. We begin with an automatically generated MR model which is 1–2 Å away from the desired structure. To this model we apply an automated refinement algorithm, seeking to obtain a fully refined structure. This puts the refinement algorithm through a rather rigorous test, especially with regard to its convergence properties. As it turns out, the *Phenix*-based procedure typically fails to produce a polished structure, *i.e.* it does not improve *R*_free_ beyond that of the initial model. In contrast, our *Amber*-based scheme consistently shows a strong performance, improving *R*_free_ to the level of the PDB-deposited structures. It is expected that in many cases, the results can be further improved by an expert manual intervention.

## 4. Discussion

The development of a structure-refinement procedure based on an MD simulation platform creates a number of potential opportunities, while also raising a number of questions. In this section, we address some of these questions and discuss some of the new possibilities.

### 4.1. Conformational diversity

A typical PDB structure consists of one or several unique protein molecules comprising the ASU. The superposition of these several molecules can be viewed as an ensemble, which is to some extent indicative of the conformational plasticity of the protein. In our case, the refinement procedure leads to multiple distinct conformational states within the UC. Likewise, the molecules in the UC can be combined into an ensemble, which is representative of conformational dynamics. The question is: how much conformational diversity is generated during a short 40 ps restrained MD run, mostly at low temperature?

**Figure 7:**
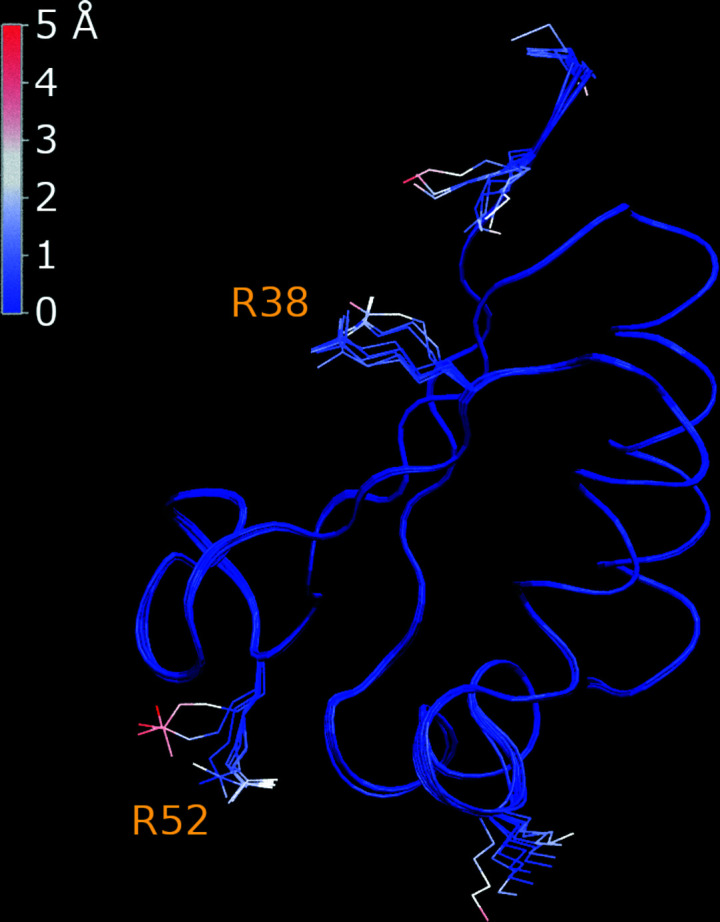
Conformational ensemble of the *Amber*-refined structure obtained from the D-model with PDB code 1dt4. Eight protein molecules in the simulated UC are superimposed via the C^α^ atoms of the secondary-structure regions. The individual structures are colored according to the deviation from the mean coordinates. Only those side chains where the deviation reaches 2.5 Å are visualized in the plot. Additionally, those side chains that sample different rotameric states (according to Lovell *et al.*, 2000 ▸) are labeled in the plot. As expected, the refined S-model displays a somewhat greater amount of conformational heterogeneity compared with the refined D-model (results not shown).

It is most convenient to address this question using those structures that contain a single protein molecule in the ASU and a large number of molecules in the UC. In our test set there are 11 structures with one protein molecule in the ASU and eight molecules in the UC. The *Amber*-generated conformational ensemble for one of these structures, PDB entry 1dt4 (Lewis *et al.*, 1999 ▸), is shown in Fig. 7 ▸. The plot represents the outcome of the refinement procedure starting from the D-model, where all protein molecules are strictly identical.

The ensemble in Fig. 7 ▸ is colored according to the deviation of the atomic coordinates in the individual molecule from the respective mean coordinates. Clearly, the refinement produced a certain amount of conformational divergence. This is particularly true for the two loops in the lower part of the plot, residues 22–24 and 39–52, where the backbone of the individual conformers deviates from the mean by as much as 1.1 Å. There are also a number of side chains that assume different rotameric states. Given that this structural ensemble retains a favorable *R*_free_ value, we assume that it reflects the actual conformational dynamics occurring in the protein crystal. In this sense, the ensemble representation is an extension of the traditional crystallographic means of modelling protein motions, such as *B* factors and alternate conformations (see below). The sampling of dynamics can be readily improved further if instead of a unit cell one uses the supercell, *i.e.* a block of multiple unit cells (Janowski *et al.*, 2016 ▸).

### 4.2. Alternate conformations

In principle, UC-based models, such as those reported in this work, or supercell-based models can obviate the need for alternate conformations. However, in most cases the dynamics leading to alternate conformations occurs on longer time scales than those sampled in the current 40 ps MD protocol. To explore the possibilities in this area, we used the structure with PDB code 3c57, which contains ten alternate side-chain conformations. The initial UC model was built using only conformation *A* for all of these side chains. This model was subjected to *Amber*-based refinement, but the net length of the restrained MD protocol was increased from 40 ps to 4 ns. As expected, during the MD run several side chains made a transition from conformation *A* to conformation *B*. Therefore, the idea of the UC model accommodating different side-chain rotameric states has been demonstrated. Of interest, such models can potentially be useful to capture collective transitions that involve concerted (correlated) rotameric jumps of two or more side chains. This also includes side-chain re­arrangements at the interfaces.

Note, however, that such extended computations are time-consuming (in the case of the 4 ns protocol, the refinement of the single structure took more than a week). Therefore, we did not pursue this line of investigation further. Our preliminary tests using long refinement protocols did not show any improvement in *R*_free_ compared with the 40 ps protocol detailed in this paper. More conclusive results in this area may be obtained after the development of a faster GPU code to calculate crystallographic forces (to be reported). Another possible option is to introduce conformational diversity into the initial model, *i.e.* populate a unit cell or a supercell with a mixture of *A* and *B* conformers.

### 4.3. Refinement of unit-cell models in *Phenix*

Of interest, the standard *Phenix* refinement protocols can be amended so as to make them more similar to our *Amber*-based approach. Specifically, one can readily construct the UC model and use it as a starting point in *Phenix* refinement, while at the same time expanding the SF data set to *P*1 and using it to drive the refinement. In the following, we refer to such an approach as the *Phenix*-*P*1 scheme. To the best of our knowledge, no one has systematically tested this scheme before. We conducted such trials using our standard test set comprised of 84 protein structures.

As it turns out, the *Phenix*-*P*1 strategy produces a significant improvement in the accuracy of the refined structures compared with the conventional *Phenix* scheme. For the refined D-models the mean *R*_free_ value (averaged over the 84 tested structures) improved from 0.258 to 0.247, whereas for the refined S-models the mean *R*_free_ improved from 0.272 to 0.269. What is the key to the success of *Phenix*-*P*1? Apparently, the enhanced conformational diversity leads to a better agreement between *F*_calc_ and *F*_obs_. This finding is in line with the previous success of ensemble-refinement methods (Burnley *et al.*, 2012 ▸; Keedy *et al.*, 2015 ▸; Levin *et al.*, 2007 ▸; Rice *et al.*, 1998 ▸). In any event, our *Amber*-based procedure retains an edge over *Phenix*-*P*1 (in particular with regard to poor initial models). Therefore, we note the potential usefulness of the *Phenix*-*P*1 scheme, but do not discuss it in the remainder of this paper.

### 4.4. Systematic absences

Our refinement procedure that operates on UC models invites another interesting option. Specifically, it is possible to add the so-called systematic absences to the experimental SF data set. Systematic absences are structure factors that are strictly equal to zero due to certain symmetry elements. In the standard symmetry-adapted models this is automatically fulfilled. However, in our approach, where the UC is treated as a *P*1 cell, the structure factors in question can deviate from zero. In this situation, systematic absences can be added to the experimental SF data set in the form of equalities *F*_obs_(*h*, *k*, *l*) = 0, providing meaningful additional restraints. This is especially relevant for nonprimitive crystal lattices (12 structures in our sample). For these structures, adding systematic absences roughly doubles the size of the *F*_obs_ data set. We chose to add all systematic absences to the work set while keeping the test set unchanged. This makes it possible to directly compare the *R*_free_ values obtained with and without the systematic absences.

Using the expanded SF data sets, we repeated *Amber*-based refinement for the 12 structures of interest. It was found that adding the systematic absences did not improve the accuracy of the refined structures. In fact, the results turned out to be somewhat worse than before. For the 12 initial models in the S-set, *R*_free_ increased on average from 0.236 to 0.241; similar values were also obtained for the D-models. We hypothesize that doubling the size of the work set through the addition of systematic absences is, in effect, equivalent to doubling the weight of the crystallographic restraints *w*_xray_. Apparently, both steps lead to some ‘overtightening’ of the structure with a concomitant slight increase in *R*_free_ (see Section 2.3). It remains to be seen whether including systematic absences and, at the same time, halving *w*_xray_ produces any improvement in the accuracy of the refined structures.

### 4.5. Using unsymmetrized data

We also experimented with another way of manipulating the SF data set. Our regular *Amber*-based protocol starts from an SF file that is fully processed and merged to high symmetry (available as part of a PDB deposition). This file is then expanded to *P*1 and used to drive the refinement. As an alternative, we employed a partially processed experimental SF file which has not been merged to high symmetry. This strategy was tested on the structure with PDB code 6sdf, for which we have the complete set of raw diffraction data (Bolgov *et al.*, 2020 ▸).

As it turns out, this altered procedure leads to somewhat less accurate results compared with our standard treatment: an *R*_free_ of 0.230 versus 0.220. Apparently, it is preferable to work with the SF data set that has been symmetrized and consequently expanded to *P*1 rather than the raw unsymmetrized data set cast as *P*1. Indeed, the symmetrization step followed by the expansion increases the number of available SF restraints (18924 versus 16234 for the data set at hand).

### 4.6. Modeling ligands

Our regular *Amber*-based refinement protocol was tested on protein structures that did not contain any ligands (see Section 2.4). In fact, it is fairly straightforward to lift this limitation. To illustrate this point, we used the same structure, PDB entry 6sdf, as mentioned above. For this structure, the UC contains 54 molecules of (4*S*)-2-methyl-2,4-pentanediol (MPD). To include these molecules in *Amber* simulations, one needs to supply the force-field parameters for the MPD molecule. In principle, these parameters can be calculated in a highly automated fashion using the program *Antechamber* (Wang *et al.*, 2006 ▸). However, we chose a more thorough approach, conducting a series of quantum-chemical calculations with *Gaussian* 16 (Frisch *et al.*, 2016 ▸).

In brief, the model MPD coordinates were downloaded from the RCSB PDB server and optimized at the DFT level of theory using the B3LYP hybrid functional (Becke, 1993 ▸) and the 6-31G* basis set (Hehre *et al.*, 1972 ▸). The electrostatic potential around the molecule was calculated on a regularly spaced grid (Janeček *et al.*, 2021 ▸) using the B3LYP functional and the aug-cc-pVDZ basis set (Dunning, 1989 ▸). The atomic charges were further calculated using the ESP scheme (Singh & Kollman, 1984 ▸). All other necessary parameters absent from the standard ff14SB force field have been adopted from the general GAFF2 field (Wang *et al.*, 2004 ▸).

Armed with the required force-field parameters, we included the 54 explicit MPD molecules in the *Amber* UC model. During the refinement we applied crystallographic forces **f** ^xray^(*x*_*j*_, *y*_*j*_, *z*_*j*_) to all MPD atoms, treating them on a par with the protein atoms. This enhanced protocol was successful, improving the *R*_free_ to 0.198 (compared with the PDB-reported value of 0.210). In the future, we intend to extend this approach to about 30 ligands that are most frequently found in the crystallographic structures (see below).

### 4.7. Comparison with PDB-REDO

The average age of the structures in our test set is 15 years. Comparing these structures with the new *Amber*-refined models may seem unfair since the refinement methodology has improved quite substantially over the last few decades. In order to address such concerns, we repeated the analysis using re-refined PDB-REDO structures for comparison. Note that PDB-REDO structures here constitute a modern alternative to the PDB structures (Joosten *et al.*, 2009 ▸, 2014 ▸). Likewise, the *Phenix*-refined coordinates used in the above analyses constitute a modern alternative to the original PDB structures. The difference is that the *Phenix* refinement protocol was designed by us (see Section 2.5), whereas the PDB-REDO structures are a given.

The comparison of *Amber*- and *Phenix*-refined structures with their PDB-REDO counterparts is summarized in Supplementary Table S4. In brief, for the initial models in the D-set *Amber* refinement improves *R*_free_ by 0.012 on average compared with the PDB-REDO structures, while also increasing the *MolProbity* score percentile from 64th to 78th. For the initial models in the S-set, the average improvement in *R*_free_ amounts to 0.005, with the *MolProbity* score percentile increasing from 64th to 76th. Although this is less impressive than the gains relative to the original PDB depositions, these results still constitute a clear-cut improvement. Hence, we conclude that the proposed *Amber*-based scheme is also competitive against the advanced refinement methodology as implemented in *PDB-REDO*.

### 4.8. PDB deposition

As an example of an *Amber*-refined UC model, we have deposited the refined version of the structure with PDB code 2msi, representing an engineered mutant of type III antifreeze protein from eelpout (DeLuca *et al.*, 1998 ▸), in the Protein Data Bank. The new model was assigned PDB code 7q3v. Compared with the original structure, it shows a substantial improvement in *R*_free_ (0.194 versus 0.261) as well as in the *MolProbity* score (96th versus 51st percentile).

## 5. Concluding remarks

The proposed refinement procedure operates on a crystal UC which is modeled as a part of the crystal lattice (*i.e.* treated as a periodic boundary box). The cell is fully solvated, including the explicitly represented bulk water. It also accommodates a certain amount of crystalline dynamics, with multiple protein molecules in the UC sampling backbone fluctuations and side-chain rotameric jumps. In addition, this model offers a highly realistic representation of crystal contacts. The evolution of the model during the refinement procedure is driven by the state-of-the-art Amber ff14SB potential *E*_force field_ and the maximum-likelihood SF-based pseudo-potential *E*_xray_.

For crystallographic structures originally classified as *P*1, the outcome of our refinement procedure is equivalent to that of the standard refinement routine (as of this date, the PDB contains 6441 such structures). Otherwise, for higher symmetry crystals the resulting UC model is distinct from the standard PDB deposition and can be viewed as a minimalistic multi-conformer ensemble. Note that the PDB currently contains close to 100 various multi-conformer X-ray structures originating from the laboratories of Brunger, Phillips, Gros, Fraser, Keedy and others. Additionally note that multi-conformer models can be seen not only as a goal in themselves, but also as a source of phase information (Rice *et al.*, 1998 ▸). The refined structures obtained by means of the new *Amber*-based protocol consistently achieve low *R*_free_ scores, comparing favorably with those reported in the PDB or attained by *Phenix*. This is illustrated in Table 1 ▸, which summarizes the results of a three-way competition between *Amber*, *Phenix* and the PDB. Clearly, *Amber*-based refinement can successfully handle even the strongly perturbed S2-models. In comparison, *Phenix*-based refinement is modestly successful when dealing with the highly accurate D-models, but becomes less competitive when applied to the scrambled models. Besides the primary *R*_free_ metric, the new *Amber* protocol also produces superior *MolProbity* scores.

One of the significant advantages of the presented refinement protocol is the absence of tunable input parameters. The calculations are started by simply pushing a button (in contrast to *Phenix*, where the user is faced with almost limitless possibilities with regard to the choice of refinement scheme). Furthermore, the calculations are reasonably fast, requiring on the order of several hours per structure on a GPU workstation. It is anticipated that the computational time will be reduced to as little as 10 min once the code has been fully optimized to take advantage of the GPU parallel architecture. Separately, it is worth noting that our refinement protocol ends with the cooling stage. Arguably, this feature approximates the cryocooling conditions during diffraction data collection.

The proposed procedure has another essential property which we see as an important advantage. Specifically, our scheme automatically balances *E*_xray_ and *E*_force field_. To explain this point, let us first consider the well structured protein scaffold, which makes the main contribution to the observed SFs. During the restrained MD run, this portion of the structure is effectively controlled (and refined) by the SF-based potential *E*_xray_. On the other hand, the mobile protein loops and tails cannot be effectively localized on the basis of the observed SFs. Hence, they are largely insensitive to *E*_xray_ and respond mainly to *E*_force field_. Finally, sites with moderate mobility (for example, those residues that act as pivots for mobile loops or tails) are controlled by a mix of *E*_xray_ and *E*_force field_. Thus, our protocol makes full use of the experimental diffraction data, with the MD machinery ‘picking up the slack’ for those regions that do not diffract well. In the grand scheme of things, this seems to be an efficient approach to crystallographic refinement.

The same logic applies to the refinement of low-resolution crystallographic structures (DeLaBarre & Brunger, 2006 ▸; DiMaio *et al.*, 2013 ▸; Schröder *et al.*, 2010 ▸). In this case the pseudo-potential *E*_xray_ is comprised of a relatively small number of SF-based restraints and therefore is relatively weak. Consequently, the balance during the refinement automatically shifts to *E*_force field_.

Parts of our code have already been ported to the official *Amber* distribution (Case *et al.*, 2020 ▸). Other elements, such as the calculation of the bulk solvent contribution, are currently being translated from *cctbx* C++ to CUDA and incorporated into *Amber* proper. Work is under way to further improve the efficiency of the GPU-based **f** ^xray^ calculations (together with S. A. Izmailov, D. S. Cerutti and D. A. Case). It should be noted, however, that GPU-equipped workstations, although fairly commonplace, are still not readily accessible to all research groups. In this sense, a designated web server offering access to the *Amber*-based refinement procedure appears to be an attractive solution. We have implemented such a pilot web server named *ARX* (*Amber-based Refinement of X-ray structures*). This server operates *Amber* under a CC BY-NC-SA 4.0 license and can be accessed at https://arx.bio-nmr.spbu.ru/.

Certain new features have been added to *ARX* compared with the treatment described in this paper. For example, in addition to proteins, the enhanced program can also work with DNA and RNA molecules. Note, however, that so far *ARX* remains a technology demonstrator rather than a solution for everyday refinement needs. In the context of this paper, *ARX* is relevant because it allows one to readily regenerate all of the *Amber*-refined models discussed above. A more detailed report on this server will be published elsewhere.

Connected to this, we have also explored other possibilities to extend the current refinement methodology.

(i) Conducting the refinement on supercells instead of unit cells.

(ii) Compiling a library of ∼30 ligands that most frequently occur in the PDB. We have estimated that with these ligands we can model and refine ∼40% of all crystallographic structures in the PDB. The force-field parameters for these ligands are either available or can be obtained using tools such as *Antechamber* (*cf.* the recently developed module *phenix.AmberPrep*; Moriarty *et al.*, 2020 ▸).

(iii) Improving the treatment of water. In principle, diffraction from ordered water molecules can be explicitly calculated during the restrained MD run. For this purpose, one needs to frequently re-identify ordered water molecules during the refinement process. This type of approach, introduced by Burnley *et al.* (2012 ▸), can be viewed as an extension of the mask-based solvent method. In principle, it is possible to go further and calculate the diffraction from all explicit water molecules contained in the (super)cell. Successful preliminary results along these lines have been obtained for a 5 × 5 × 5 supercell of tetragonal lysozyme (N. Liu, N. R. Skrynnikov & Y. Xue, to be published).

(iv) A specialized application to refine mobile loops. As indicated above, the proposed scheme is well suited to refine mobile elements of the protein structure, fully utilizing the structural information encoded in the SF data, while relying on a high-quality force field to ‘fill the gaps’. The initial loop conformations can be built using existing programs such as the *Rosetta* loop-reconstruction module (Mandell *et al.*, 2009 ▸), the *MODELLER* loop-reconstruction module (Fiser *et al.*, 2000 ▸), *RCD*+ (López-Blanco *et al.*, 2016 ▸), *FREAD* (Choi & Deane, 2010 ▸), *DaReUS-Loop* (Karami *et al.*, 2018 ▸) or others. The resulting models will then be refined using the same principles as described in this paper.

(v) The development of more accurate MD models for protein crystals. Restrained trajectories, such as discussed in this paper, offer a path towards improved MD models of protein crystals (Xue & Skrynnikov, 2014 ▸). In this case, *E*_xray_ can be viewed as an empirical potential which compensates for the shortcomings of the conventional force fields (Raval *et al.*, 2012 ▸).

Bringing together high-resolution X-ray diffraction data and state-of-the-art MD engines should lead to a valuable synergy and eventually pay some dividend, especially with regard to more mobile elements of the structure. The implementation of this concept, however, has been a challenge and progress thus far has been incremental. The advent of GPU computing has opened new possibilities in this area. In particular, the *Amber* program offers a good platform for solving biomolecular structures. Of note, *Amber* is equipped with well developed modules to calculate NMR structures. For certain applications, such as oligonucleotide structure determination by NMR, it is reputed to be the best of all existing software options. As demonstrated in this paper, *Amber* can also be used as an efficient platform for the refinement of crystallo­graphic structures. Further progress in this direction should create new opportunities in the area of structural crystallo­graphy, as well as in cryogenic electron microscopy and other emerging techniques to probe biomolecular structure and dynamics.

## Supplementary Material

PDB reference: type III antifreeze protein from eelpout, 7q3v

Summary of equations and Supplementary Tables,. DOI: 10.1107/S2052252521011891/lz5053sup1.pdf

## Figures and Tables

**Table 1:** The number of first-place finishes, as judged by the lowest *R*_free_, in a three-way competition between *Amber*-refined, *Phenix*-refined and PDB-deposited structures The results are from the test set including 74 protein structures (see Section 2.4).

Initial models	R.m.s.d. from PDB-deposited structures (Å)	No. of refined structures with lowest *R*_free_		
		*Amber*	*Phenix*	PDB
D-set	0	42	17	15
S-set	1.06	46	6	22
S1-set	1.32	35	9	30
S2-set	1.55	27	10	37
