# Supplementary material for: Modeling a unit cell: crystallographic refinement procedure using the biomolecular MD simulation platform Amber
Source: IUCrJ. 2021 Dec 16;9(Pt 1):114–33. doi: 10.1107/S2052252521011891 (PMC8733891; doi:10.1107/S2052252521011891)
Supplement: Supplementary file 1 [file m-09-00114-sup1.pdf]

IUCrJ

Volume 9 (2022)

Supporting information for article:

Modeling a unit cell: crystallographic refinement procedure using the biomolecular MD simulation platform *Amber*

Oleg Mikhailovskii, Yi Xue and Nikolai R. Skrynnikov

Supplementary Information

Modeling a unit cell: crystallographic refinement procedure using the biomolecular MD simulation platform Amber

Oleg Mikhailovskii,[1,2] Yi Xue[3-5] and Nikolai R. Skrynnikov[1,2]✉

[1] Laboratory of Biomolecular NMR, St. Petersburg State University, St. Petersburg 199034, Russia

[2] Department of Chemistry, Purdue University, West Lafayette, Indiana 47907, United States

[3] School of Life Sciences, Tsinghua University, Beijing 100084, China

[4] Beijing Advanced Innovation Center for Structural Biology, Tsinghua University, Beijing 100084, China

[5] Tsinghua University - Peking University Joint Center for Life Sciences, Tsinghua University, Beijing 100084, China

✉ n.skrynnikov@spbu.ru

Summary of equations

$$\mathbf{F}_{calc}^{protein}(h,k,l) = \sum_{n=1}^{N_{atoms}} q_n f_n(s) \exp\left(-\frac{B_n s^2}{4}\right) \exp(2\pi i \vec{r}_n \vec{s}) \quad \text{(S1)}$$

where $\mathbf{F}_{calc}^{protein}(h,k,l)$ is the calculated structure factor originating from the protein atoms, $\{h,k,l\}$ is the set of Miller indices, the summation is over the entire set of protein atoms (total number of atoms N_{atoms}), q_n is the atomic occupancy (assumed 1.0 in this work), $f_n(s)$ are atomic form-factors (calculated according to it1992 table, which ensures high accuracy for crystallographic structures with resolution >1.5 Å; in our implementation, $f_n(s)$ are pre-calculated for the entire set of reflections which achieves a significant speed-up in the calculations using Eq. (S1)), $\vec{s}$ is the vector in the reciprocal space with the norm s, B_n is the atomic B factor, and $\vec{r}_n$ is the vector of atomic coordinates. Here and in what follows we use the Cartesian coordinates (rather than fractional coordinates).

$$\mathbf{F}_{calc}(h,k,l) = k_{overall} k_{iso} k_{aniso} \left(\mathbf{F}_{calc}^{protein}(h,k,l) + k_{mask} \mathbf{F}_{calc}^{bulk\ solvent}(h,k,l)\right) \quad \text{(S2)}$$

where $\mathbf{F}_{calc}(h,k,l)$ is the calculated structure factor originating from both protein atoms and interstitial water, $k_{overall}$ is the scaling factor to normalize the arbitrary SF scale, $k_{iso}$and k_{aniso} are (resolution-shell-dependent) scaling factors due to isotropic and anisotropic lattice vibrations, k_{mask} is a (resolution-shell-dependent) bulk-solvent scaling factor and $\mathbf{F}_{calc}^{bulk\ solvent}(h,k,l)$ is the mask-based bulk-solvent contribution into the calculated SFs.

$$E_{xray} = \sum_{\{h,k,l\}} \left(-\ln\left(\frac{2F_{obs}(h,k,l)}{\varepsilon\beta}\right) + \frac{F_{obs}^2(h,k,l)}{\varepsilon\beta} + \frac{\alpha^2 F_{calc}^2(h,k,l)}{\varepsilon\beta} - \ln I_0\left(\frac{2\alpha F_{obs}(h,k,l) F_{calc}(h,k,l)}{\varepsilon\beta}\right)\right) \quad \text{(S3)}$$

where E_{xray} is the ML-based pseudo-energy, the summation is over all reflections, $F_{obs}(h,k,l)$ are the amplitudes of experimental SFs, $F_{calc}(h,k,l)$ are the amplitudes of calculated SFs, α and β are (resolution-shell-dependent) ML likelihood distribution parameters, ε is the symmetry coefficient ($\varepsilon = 1$ for the space group P1 at hand), and $I_0(x)$ is the zeroth-order modified Bessel function of the first kind.

$$f_x^{xray}(x_j, y_j, z_j) = -w_{xray} \frac{\partial E_{xray}}{\partial x_j}$$

$$f_y^{xray}(x_j, y_j, z_j) = -w_{xray} \frac{\partial E_{xray}}{\partial y_j} \quad \text{(S4.1)}$$

$$f_z^{xray}(x_j, y_j, z_j) = -w_{xray} \frac{\partial E_{xray}}{\partial z_j}$$

$$\frac{\partial E_{xray}}{\partial x_j} = \sum_{\{h,k,l\}} \kappa \mathrm{Re}\left\{\frac{\partial \mathbf{F}_{calc}(h,k,l)}{\partial x_j} \mathbf{F}_{calc}^*(h,k,l)\right\} \left(\alpha - \frac{F_{obs}(h,k,l)}{F_{calc}(h,k,l)} \frac{I_1(\kappa F_{obs}(h,k,l) F_{calc}(h,k,l))}{I_0(\kappa F_{obs}(h,k,l) F_{calc}(h,k,l))}\right)$$

$$\frac{\partial E_{xray}}{\partial y_j} = \sum_{\{h,k,l\}} \kappa \mathrm{Re}\left\{\frac{\partial \mathbf{F}_{calc}(h,k,l)}{\partial y_j} \mathbf{F}_{calc}^*(h,k,l)\right\} \left(\alpha - \frac{F_{obs}(h,k,l)}{F_{calc}(h,k,l)} \frac{I_1(\kappa F_{obs}(h,k,l) F_{calc}(h,k,l))}{I_0(\kappa F_{obs}(h,k,l) F_{calc}(h,k,l))}\right) \quad \text{(S4.2)}$$

$$\frac{\partial E_{xray}}{\partial z_j} = \sum_{\{h,k,l\}} \kappa \mathrm{Re}\left\{\frac{\partial \mathbf{F}_{calc}(h,k,l)}{\partial z_j} \mathbf{F}_{calc}^*(h,k,l)\right\} \left(\alpha - \frac{F_{obs}(h,k,l)}{F_{calc}(h,k,l)} \frac{I_1(\kappa F_{obs}(h,k,l) F_{calc}(h,k,l))}{I_0(\kappa F_{obs}(h,k,l) F_{calc}(h,k,l))}\right)$$

$$\frac{\partial \mathbf{F}_{calc}(h,k,l)}{\partial x_j} = (2\pi i)k_{overall}k_{iso}k_{aniso}q_j f_j(s)\exp\left(-\frac{B_j s^2}{4}\right)(hb_{1x} + kb_{2x} + lb_{3x})\exp\left(2\pi i\vec{r}_j\vec{s}\right)$$

$$\frac{\partial \mathbf{F}_{calc}(h,k,l)}{\partial y_j} = (2\pi i)k_{overall}k_{iso}k_{aniso}q_j f_j(s)\exp\left(-\frac{B_j s^2}{4}\right)\left(hb_{1y} + kb_{2y} + lb_{3y}\right)\exp\left(2\pi i\vec{r}_j\vec{s}\right) \quad \text{(S4.3)}$$

$$\frac{\partial \mathbf{F}_{calc}(h,k,l)}{\partial z_j} = (2\pi i)k_{overall}k_{iso}k_{aniso}q_j f_j(s)\exp\left(-\frac{B_j s^2}{4}\right)(hb_{1z} + kb_{2z} + lb_{3z})\exp\left(2\pi i\vec{r}_j\vec{s}\right)$$

Here index *j* enumerates the *j*-th atom, $\kappa = 2\alpha/\varepsilon\beta$, $I_1(x)$ is the first-order modified Bessel function of the first kind, and vectors $\vec{b}_1$, $\vec{b}_2$ and $\vec{b}_3$ are the primitive vectors of the reciprocal lattice

$$\vec{b}_1 = \frac{\vec{a}_2\times\vec{a}_3}{\vec{a}_1\cdot(\vec{a}_2\times\vec{a}_3)}$$

$$\vec{b}_2 = \frac{\vec{a}_3\times\vec{a}_1}{\vec{a}_1\cdot(\vec{a}_2\times\vec{a}_3)} \quad \text{(S5)}$$

$$\vec{b}_3 = \frac{\vec{a}_1\times\vec{a}_2}{\vec{a}_1\cdot(\vec{a}_2\times\vec{a}_3)}$$

$$\vec{s} = h\vec{b}_1 + k\vec{b}_2 + l\vec{b}_3 \quad \text{(S6)}$$

with vectors $\vec{a}_1$, $\vec{a}_2$ and $\vec{a}_3$ being the primitive vectors of the direct lattice. In relation to Eq. (S4) one should bear in mind that k_{iso}, k_{aniso}, α and β are resolution-shell-dependent.

Table S1

Protein structures used to test the new *Amber*-based refinement procedure

PDB code	Resolution*	Ordered water	R_{work}	R_{free}	Space group
5lhx	1.53	35	0.167	0.209	P 1 21 1
4f17	1.55	45	0.200	0.217	P 21 21 21
4x37	1.63	43	0.198	0.229	P 21 21 21
6msi	1.65	0	–	–	P 21 21 21
1ame	1.65	45	0.199	0.282	P 21 21 21
1ae2	1.65	48	0.223	0.326	C 1 2 1
3le4	1.70	36	0.181	0.213	P 43 21 2
2fht	1.70	27	0.239	0.331	P 41 2 2
1loz	1.80	43	0.211	–	P 21 21 21
1yib	1.80	41	0.221	0.259	C 2 2 21
3zye	1.85	34	0.222	0.258	C 1 2 1
1ail	1.90	34	0.182	0.229	P 43 21 2
2msi	1.90	0	0.193	0.261	P 21 21 21
5f6a	1.92	29	0.223	0.250	C 1 2 1
3tsv	1.99	32	0.269	0.315	P 21 21 21
1mjc	2.00	36	–	–	P 21 21 21
1ae3	2.00	47	0.181	0.284	C 1 2 1
4c86	2.00	29	0.200	0.276	P 1 21 1
1x6j	2.00	37	0.188	0.242	I 2 2 2
4f26	2.00	9	0.216	0.286	C 2 2 21
1q2y	2.03	36	0.234	0.265	P 21 21 21
4qfq	2.04	14	0.230	0.287	P 21 21 21
4wfw	2.05	15	0.209	0.254	P 43 21 2
5teo	2.05	15	0.201	0.244	P 1 21 1
1z27	2.08	21	0.258	0.267	P 21 21 21
1bkl	2.09	39	0.200	0.304	P 21 21 2
5a7l	2.10	39	0.216	0.236	P 21 21 2
2nn4	2.10	47	0.247	0.280	P 1 21 1
171l	2.11	0	–	–	P 21 21 2
1smt	2.12	0	0.217	0.250	P 1 21 1
3fis	2.13	29	0.183	–	P 21 21 21
3zy1	2.15	0	0.236	0.285	P 4 2 2
1anu	2.15	24	0.197	0.219	C 1 2 1
2o85	2.17	40	0.196	0.245	P 21 21 21
1kem	2.19	0	0.183	0.288	P 1
5t8n	2.20	13	0.243	0.345	P 1 21 1
5t8l	2.20	47	0.191	0.259	P 1 21 1

4hll	2.20	34	0.211	0.233	C 1 2 1
6cyr	2.20	26	0.204	0.249	P 21 21 21
1k40	2.20	24	0.236	0.294	C 1 2 1
1wdx	2.20	16	0.263	0.326	P 1
4fis	2.20	32	0.185	–	P 21 21 21
5xbh	2.23	47	0.229	0.281	P 1
5fd7	2.27	7	0.259	0.263	P 1 21 1
2ont	2.30	19	0.243	0.294	P 43 21 2
4niq	2.30	0	0.236	0.257	P 21 21 21
3u4z	2.30	46	0.220	0.266	P 1 21 1
3qd7	2.30	21	0.224	0.244	P 21 21 2
2eql	2.33	0	0.234	–	P 21 21 21
5ewr	2.35	42	0.166	0.220	P 21 21 21
2o87	2.40	11	0.193	0.259	P 21 21 21
2aak	2.40	0	0.221	0.287	P 21 21 21
1o9h	2.40	29	0.209	0.241	P 1 21 1
3rd3	2.40	49	0.217	0.295	P 1 21 1
149l	2.45	38	–	–	P 21 21 21
2fkl	2.49	19	0.222	0.263	P 1 21 1
2qdo	2.50	18	0.248	0.279	P 1
3q2c	2.50	32	0.225	0.257	C 1 2 1
1bmg	2.50	24	–	–	P 21 21 21
1rn7	2.50	32	0.191	0.229	P 21 21 2
3dvp	2.50	49	0.204	0.263	P 21 21 21
1du5	2.50	39	0.167	0.258	P 1 21 1
1w45	2.51	27	0.206	0.269	P 1
3zq7	2.52	23	0.236	0.283	P 43 21 2
2o89	2.55	0	0.202	0.255	P 21 21 21
1dt4	2.60	0	0.222	0.282	P 42 21 2
1uue	2.60	24	0.240	0.266	P 21 21 21
5tut	2.60	0	0.200	0.243	P 1 21 1
4oyc	2.60	7	0.227	0.259	C 1 2 1
2snw	2.68	12	0.191	–	P 1 21 1
3ndf	2.70	22	0.211	0.268	P 1 21 1
5h79	2.70	17	0.230	0.278	P 1 21 1
3m3t	2.79	13	0.185	0.273	P 1 21 1
3hpm	2.80	1	0.243	0.295	P 21 21 21
4bhc	2.80	48	0.176	0.223	P 21 21 2
4c0m	2.80	0	0.252	0.291	P 1
3k9p	2.80	0	0.232	0.296	P 1 21 1
2jee	2.80	24	0.310	0.321	P 1
4ug3	2.80	2	0.216	0.301	P 1 21 1

5arj	2.90	0	0.207	0.282	P 1
2j7i	2.90	31	0.248	0.295	P 21 21 21
6dz6	3.01	14	0.224	0.283	P 21 21 21
2nsb	3.20	0	0.256	0.262	P 21 21 2
5jqz	3.83	0	0.428	0.479	P 21 21 2

* As reported by *MolProbity* based on the analyses of the SF data.

Table S2

Success rate of different *Phenix* refinement protocols. Number of "wins" for each individual protocol, where the win corresponds to the best (lowest R_{free}) refined model in the pool of 32 competing *Phenix* models. The list of abbreviations: CDL – conformation dependent library, Amb - Amber ff14SB, SA – simulated annealing, TAD – torsional angle dynamics, CC – Cartesian coordinates, WO – weight optimization, BO – B-factors optimization (see section 2.5 in the main text for further details).

**S-set**				
CDL	SA-TAD-CC	WO	no BO	11
CDL	SA-TAD-CC	no WO	no BO	9
CDL	SA-CC	no WO	BO	8
CDL	SA-CC	WO	no BO	8
CDL	SA-TAD-CC	no WO	BO	7
Amb	SA-TAD	no WO	no BO	7
CDL	SA-CC	no WO	no BO	5
CDL	SA-CC	WO	BO	4
Amb	SA-TAD	no WO	BO	4
CDL	SA-TAD	no WO	no BO	4
Amb	no SA	no WO	BO	3
Amb	no SA	no WO	no BO	3
CDL	SA-TAD-CC	WO	BO	2
CDL	SA-TAD	no WO	BO	2
CDL	no SA	WO	BO	1
CDL	no SA	no WO	BO	1
Amb	no SA	WO	BO	1
Amb	SA-TAD	WO	BO	1
CDL	no SA	WO	no BO	1
CDL	no SA	no WO	no BO	1
Amb	SA-TAD	WO	no BO	1

**D-set**				
CDL	no SA	WO	no BO	26
CDL	no SA	WO	BO	9
Amb	no SA	no WO	no BO	6
CDL	no SA	no WO	no BO	5
Amb	no SA	WO	no BO	4
CDL	SA-TAD	WO	no BO	4
CDL	no SA	no WO	BO	3
CDL	SA-TAD-CC	no WO	BO	3
CDL	SA-TAD-CC	WO	no BO	3
CDL	SA-CC	no WO	no BO	3
Amb	no SA	WO	BO	2
Amb	no SA	no WO	BO	2
CDL	SA-CC	no WO	BO	2
CDL	SA-TAD	WO	BO	2
CDL	SA-TAD-CC	no WO	no BO	2
CDL	SA-CC	WO	no BO	2
Amb	SA-TAD	no WO	no BO	2
CDL	SA-CC	WO	BO	1
Amb	SA-TAD	no WO	BO	1
Amb	SA-TAD	WO	BO	1
CDL	SA-TAD	no WO	no BO	1

Table S3

Refinement statistics for *Amber* and *Phenix* refinement of 84 protein structures (S- and D-models)

PDB code	R_{free} (PDB structures)	R_{free} (*Amber*-refined S-models)	R_{free} (*Phenix*-refined S-models)	R_{free} (*Amber*-refined D-models)	R_{free} (*Phenix*-refined D-models)	*MolProbity* percentile (PDB structures)	*MolProbity* percentile (*Amber*-refined S-models)	*MolProbity* percentile (*Phenix*-refined S-models)	*MolProbity* percentile (*Amber*-refined D-models)	*MolProbity* percentile (*Phenix*-refined D-models)
5lhx	0.209	0.233	0.222	0.226	0.208	83.07	48.81	34.04	83.92	51.92
4f17	0.217	0.209	0.236	0.203	0.234	30.85	64.87	23.71	76.86	89.43
4x37	0.229	0.200	0.225	0.200	0.217	7.46	33.18	19.16	42.01	26.01
6msi	–	0.193	0.206	0.182	0.206	82.20	95.92	65.91	90.62	82.20
1ame	0.282	0.194	0.219	0.186	0.226	90.62	38.12	30.01	62.03	90.62
1ae2	0.326	0.236	0.216	0.233	0.220	5.93	69.51	10.32	23.67	78.53
3le4	0.213	0.191	0.192	0.187	0.201	89.37	86.10	71.77	99.50	99.69
2fht	0.331	0.333	0.322	0.305	0.304	8.34	8.81	7.43	22.07	22.07
1loz	–	0.190	0.210	0.190	0.209	16.77	63.37	54.45	82.40	70.28
1yib	0.259	0.216	0.236	0.204	0.237	53.64	61.56	8.74	66.95	46.78
3zye	0.258	0.234	0.254	0.228	0.235	4.72	55.36	11.60	44.85	21.51
1ail	0.229	0.190	0.233	0.198	0.214	63.10	98.94	57.10	97.94	95.33
2msi	0.261	0.204	0.219	0.194	0.212	51.45	77.36	68.40	95.60	44.25
5f6a	0.250	0.240	0.273	0.246	0.250	62.48	84.98	13.81	89.91	50.52
3tsv	0.315	0.290	0.312	0.287	0.292	7.82	36.54	4.81	19.35	5.91
1mjc	–	0.209	0.205	0.208	0.200	15.91	74.58	14.19	58.17	39.28
1ae3	0.284	0.195	0.210	0.197	0.210	13.88	58.85	71.42	53.60	94.52
4c86	0.276	0.242	0.259	0.235	0.237	20.53	66.11	9.21	81.48	15.91
1x6j	0.242	0.231	0.268	0.223	0.242	35.49	75.25	42.84	74.58	83.87
4f26	0.286	0.226	0.272	0.219	0.263	29.16	68.73	61.26	72.12	48.09
1q2y	0.265	0.228	0.240	0.222	0.233	16.55	66.42	27.99	67.74	74.85
4qfq	0.287	0.260	0.276	0.251	0.263	11.73	38.21	17.25	33.80	19.20
4wfw	0.254	0.277	0.294	0.272	0.293	33.93	92.38	42.05	94.34	39.59
5teo	0.244	0.226	0.241	0.219	0.226	97.39	90.21	49.75	81.43	31.05
1z27	0.267	0.304	0.305	0.268	0.263	8.84	20.55	3.71	58.33	9.06

1bkl	0.304	0.279	0.280	0.276	0.270	3.70	23.08	2.63	16.87	5.07
5a7l	0.236	0.244	0.254	0.241	0.244	99.79	85.53	70.08	95.95	70.70
2nn4	0.280	0.261	0.273	0.253	0.267	10.01	69.34	57.17	57.17	52.13
171l	–	0.285	0.293	0.277	0.292	1.27	86.74	11.49	77.96	17.65
1smt	0.250	0.234	0.233	0.226	0.223	66.14	81.82	82.28	81.31	64.31
3fis	–	0.235	0.230	0.233	0.228	7.27	61.99	8.31	58.52	27.54
3zy1	0.285	0.264	0.310	0.264	0.312	79.73	98.75	6.48	95.12	24.26
1anu	0.219	0.198	0.207	0.198	0.203	63.63	86.64	89.51	75.51	50.62
2o85	0.245	0.217	0.227	0.210	0.233	67.40	65.58	77.98	86.87	80.03
1kem	0.288	0.245	0.265	0.231	0.251	4.98	63.75	10.28	56.41	18.51
5t8n	0.345	0.284	0.332	0.283	0.298	67.23	93.22	8.58	87.14	52.25
5t8l	0.259	0.235	0.263	0.232	0.246	79.19	88.81	62.74	98.30	47.59
4hll	0.233	0.228	0.241	0.232	0.238	14.31	86.79	58.78	85.46	79.70
6cyr	0.249	0.248	0.244	0.244	0.241	79.70	81.73	26.14	77.77	25.12
1k40	0.294	0.235	0.223	0.222	0.217	9.60	94.69	64.51	81.73	99.90
1wdx	0.326	0.321	0.339	0.306	0.295	52.25	51.67	3.47	89.68	43.57
4fis	–	0.247	0.247	0.235	0.240	5.45	68.33	32.38	58.10	24.64
5xbh	0.281	0.255	0.266	0.249	0.244	97.13	66.32	54.71	88.81	75.50
5fd7	0.263	0.324	0.366	0.332	0.331	10.80	67.59	6.49	75.60	61.94
2ont	0.294	0.335	0.340	0.320	0.342	7.08	60.19	34.56	66.74	6.87
4niq	0.257	0.300	0.307	0.286	0.289	16.65	73.42	12.06	70.90	47.01
3u4z	0.266	0.257	0.261	0.247	0.251	7.82	47.64	17.05	36.16	19.27
3qd7	0.244	0.234	0.256	0.231	0.241	38.38	88.25	66.29	81.67	84.94
2eql	–	0.301	0.303	0.290	0.285	4.78	41.91	11.34	48.73	22.79
5ewr	0.220	0.231	0.233	0.226	0.223	69.27	99.73	99.47	97.80	99.47
2o87	0.259	0.218	0.225	0.211	0.230	70.32	84.92	65.65	94.61	55.71
2aak	0.287	0.230	0.236	0.227	0.238	16.05	78.60	61.33	76.60	81.31
1o9h	0.241	0.234	0.259	0.228	0.236	54.60	89.56	24.69	92.40	71.33
3rd3	0.295	0.271	0.294	0.270	0.274	35.56	87.25	84.30	89.15	68.49
149l	–	0.223	0.219	0.220	0.217	4.57	97.90	75.48	93.06	93.56
2fkl	0.263	0.254	0.293	0.249	0.269	32.47	75.58	30.58	71.75	44.64
2qdo	0.279	0.279	0.285	0.270	0.277	4.92	66.41	20.86	58.11	27.03
3q2c	0.257	0.276	0.293	0.280	0.295	15.29	92.27	84.77	95.12	14.10
1bmg	–	0.235	0.236	0.228	0.231	30.03	87.12	74.45	86.82	33.68

1rn7	0.229	0.225	0.231	0.216	0.214	63.19	60.43	31.02	67.41	65.53
3dvp	0.263	0.269	0.278	0.260	0.260	21.82	65.53	87.76	73.92	63.61
1du5	0.258	0.223	0.224	0.205	0.200	60.43	61.29	30.03	84.02	81.71
1w45	0.269	0.246	0.267	0.245	0.235	35.51	80.26	15.73	64.00	42.08
3zq7	0.283	0.297	0.313	0.295	0.292	6.08	60.77	17.31	45.28	12.11
2o89	0.255	0.222	0.215	0.221	0.248	47.02	83.72	93.52	91.18	70.91
1dt4	0.282	0.282	0.284	0.280	0.284	20.98	87.33	32.34	90.64	13.81
1uue	0.266	0.240	0.251	0.238	0.244	21.42	94.83	74.58	82.09	56.91
5tut	0.243	0.257	0.253	0.249	0.236	99.85	94.43	44.28	97.18	55.39
4oyc	0.259	0.320	0.334	0.309	0.296	58.91	74.58	58.91	87.60	42.37
2snw	–	0.225	0.244	0.210	0.228	23.54	97.90	83.26	96.06	77.45
3ndf	0.268	0.253	0.258	0.247	0.250	46.89	96.50	44.43	97.19	78.73
5h79	0.278	0.309	0.315	0.306	0.302	86.98	93.28	8.38	91.36	39.53
3m3t	0.273	0.249	0.272	0.254	0.253	40.95	99.22	76.98	96.79	92.82
3hpm	0.295	0.308	0.327	0.292	0.290	27.84	89.78	6.27	80.14	82.58
4bhc	0.223	0.249	0.274	0.258	0.242	55.00	99.21	70.69	99.63	78.36
4c0m	0.291	0.318	0.355	0.317	0.314	92.11	99.91	87.52	100.00	79.74
3k9p	0.296	0.277	0.315	0.260	0.255	11.71	97.71	7.28	96.97	43.24
2jee	0.321	0.404	0.398	0.377	0.332	15.07	87.18	2.15	96.05	16.06
4ug3	0.301	0.277	0.316	0.265	0.278	89.78	98.77	37.12	99.59	91.90
5arj	0.282	0.283	0.349	0.258	0.280	87.87	86.49	4.70	97.98	55.42
2j7i	0.295	0.296	0.318	0.285	0.297	20.49	98.75	32.01	97.73	38.90
6dz6	0.283	0.294	0.303	0.289	0.306	93.24	99.80	43.17	99.50	51.23
2nsb	0.262	0.286	0.292	0.270	0.269	36.34	98.75	97.46	95.30	83.95
5jqz	0.479	0.471	0.610	0.460	0.592	91.86	99.22	37.09	99.22	31.30

Table S4

Refinement statistics for *Amber* and *Phenix* refinement of 84 protein structures (S- and D-models) compared to PDB-REDO

PDB code	R_{free} (PDB-REDO structures)	R_{free} (*Amber*-refined S-models)	R_{free} (*Phenix*-refined S-models)	R_{free} (*Amber*-refined D-models)	R_{free} (*Phenix*-refined D-models)	*MolProbity* percentile (PDB-REDO structures)	*MolProbity* percentile (*Amber*-refined S-models)	*MolProbity* percentile (*Phenix*-refined S-models)	*MolProbity* percentile (*Amber*-refined D-models)	*MolProbity* percentile (*Phenix*-refined D-models)
5lhx	0.221	0.233	0.222	0.226	0.208	86.17	48.81	34.04	83.92	51.92
4f17	0.181	0.209	0.236	0.203	0.234	41.06	64.87	23.71	76.86	89.43
4x37	0.232	0.200	0.225	0.200	0.217	46.65	33.18	19.16	42.01	26.01
6msi	0.226	0.193	0.206	0.182	0.206	62.93	95.92	65.91	90.62	82.20
1ame	0.208	0.194	0.219	0.186	0.226	89.42	38.12	30.01	62.03	90.62
1ae2	0.233	0.236	0.216	0.233	0.220	34.86	69.51	10.32	23.67	78.53
3le4	0.191	0.191	0.192	0.187	0.201	71.77	86.10	71.77	99.50	99.69
2fht	0.280	0.333	0.322	0.305	0.304	73.50	8.81	7.43	22.07	22.07
1loz	0.231	0.190	0.210	0.190	0.209	92.51	63.37	54.45	82.40	70.28
1yib	0.214	0.216	0.236	0.204	0.237	99.37	61.56	8.74	66.95	46.78
3zye	0.230	0.234	0.254	0.228	0.235	18.27	55.36	11.60	44.85	21.51
1ail	0.255	0.190	0.233	0.198	0.214	46.47	98.94	57.10	97.94	95.33
2msi	0.195	0.204	0.219	0.194	0.212	91.92	77.36	68.40	95.60	44.25
5f6a	0.254	0.240	0.273	0.246	0.250	88.97	84.98	13.81	89.91	50.52
3tsv	0.295	0.290	0.312	0.287	0.292	8.49	36.54	4.81	19.35	5.91
1mjc	0.226	0.209	0.205	0.208	0.200	46.07	74.58	14.19	58.17	39.28
1ae3	0.266	0.195	0.210	0.197	0.210	41.60	58.85	71.42	53.60	94.52
4c86	0.246	0.242	0.259	0.235	0.237	86.24	66.11	9.21	81.48	15.91
1x6j	0.214	0.231	0.268	0.223	0.242	44.64	75.25	42.84	74.58	83.87
4f26	0.221	0.226	0.272	0.219	0.263	68.73	68.73	61.26	72.12	48.09
1q2y	0.247	0.228	0.240	0.222	0.233	91.36	66.42	27.99	67.74	74.85
4qfq	0.254	0.260	0.276	0.251	0.263	31.42	38.21	17.25	33.80	19.20
4wfw	0.244	0.277	0.294	0.272	0.293	99.82	92.38	42.05	94.34	39.59
5teo	0.257	0.226	0.241	0.219	0.226	21.62	90.21	49.75	81.43	31.05
1z27	0.272	0.304	0.305	0.268	0.263	68.43	20.55	3.71	58.33	9.06

1bkl	0.262	0.279	0.280	0.276	0.270	6.10	23.08	2.63	16.87	5.07
5a7l	0.295	0.244	0.254	0.241	0.244	62.72	85.53	70.08	95.95	70.70
2nn4	0.265	0.261	0.273	0.253	0.267	60.81	69.34	57.17	57.17	52.13
171l	0.328	0.285	0.293	0.277	0.292	77.44	86.74	11.49	77.96	17.65
1smt	0.230	0.234	0.233	0.226	0.223	88.79	81.82	82.28	81.31	64.31
3fis	0.235	0.235	0.230	0.233	0.228	39.32	61.99	8.31	58.52	27.54
3zy1	0.181	0.264	0.310	0.264	0.312	78.18	98.75	6.48	95.12	24.26
1anu	0.286	0.198	0.207	0.198	0.203	67.68	86.64	89.51	75.51	50.62
2o85	0.197	0.217	0.227	0.210	0.233	57.83	65.58	77.98	86.87	80.03
1kem	0.297	0.245	0.265	0.231	0.251	20.19	63.75	10.28	56.41	18.51
5t8n	0.332	0.284	0.332	0.283	0.298	90.89	93.22	8.58	87.14	52.25
5t8l	0.232	0.235	0.263	0.232	0.246	85.46	88.81	62.74	98.30	47.59
4hll	0.268	0.228	0.241	0.232	0.238	59.95	86.79	58.78	85.46	79.70
6cyr	0.248	0.248	0.244	0.244	0.241	85.46	81.73	26.14	77.77	25.12
1k40	0.255	0.235	0.223	0.222	0.217	81.73	94.69	64.51	81.73	99.90
1wdx	0.268	0.321	0.339	0.306	0.295	15.83	51.67	3.47	89.68	43.57
4fis	0.282	0.247	0.247	0.235	0.240	95.75	68.33	32.38	58.10	24.64
5xbh	0.280	0.255	0.266	0.249	0.244	63.65	66.32	54.71	88.81	75.50
5fd7	0.260	0.324	0.366	0.332	0.331	80.80	67.59	6.49	75.60	61.94
2ont	0.237	0.335	0.340	0.320	0.342	48.77	60.19	34.56	66.74	6.87
4niq	0.298	0.300	0.307	0.286	0.289	24.59	73.42	12.06	70.90	47.01
3u4z	0.229	0.257	0.261	0.247	0.251	79.78	47.64	17.05	36.16	19.27
3qd7	0.352	0.234	0.256	0.231	0.241	92.08	88.25	66.29	81.67	84.94
2eql	0.222	0.301	0.303	0.290	0.285	99.46	41.91	11.34	48.73	22.79
5ewr	0.228	0.231	0.233	0.226	0.223	78.05	99.73	99.47	97.80	99.47
2o87	0.241	0.218	0.225	0.211	0.230	71.33	84.92	65.65	94.61	55.71
2aak	0.324	0.230	0.236	0.227	0.238	17.82	78.60	61.33	76.60	81.31
1o9h	0.246	0.234	0.259	0.228	0.236	67.44	89.56	24.69	92.40	71.33
3rd3	0.241	0.271	0.294	0.270	0.274	63.53	87.25	84.30	89.15	68.49
149l	0.201	0.223	0.219	0.220	0.217	82.55	97.90	75.48	93.06	93.56
2fkl	0.262	0.254	0.293	0.249	0.269	70.22	75.58	30.58	71.75	44.64
2qdo	0.220	0.279	0.285	0.270	0.277	67.41	66.41	20.86	58.11	27.03
3q2c	0.274	0.276	0.293	0.280	0.295	17.89	92.27	84.77	95.12	14.10
1bmg	0.283	0.235	0.236	0.228	0.231	8.71	87.12	74.45	86.82	33.68

1rn7	0.309	0.225	0.231	0.216	0.214	73.92	60.43	31.02	67.41	65.53
3dvp	0.227	0.269	0.278	0.260	0.260	65.53	65.53	87.76	73.92	63.61
1du5	0.226	0.223	0.224	0.205	0.200	29.47	61.29	30.03	84.02	81.71
1w45	0.327	0.246	0.267	0.245	0.235	15.46	80.26	15.73	64.00	42.08
3zq7	0.240	0.297	0.313	0.295	0.292	82.17	60.77	17.31	45.28	12.11
2o89	0.267	0.222	0.215	0.221	0.248	60.30	83.72	93.52	91.18	70.91
1dt4	0.293	0.282	0.284	0.280	0.284	84.58	87.33	32.34	90.64	13.81
1uue	0.324	0.240	0.251	0.238	0.244	41.30	94.83	74.58	82.09	56.91
5tut	0.285	0.257	0.253	0.249	0.236	68.92	94.43	44.28	97.18	55.39
4oyc	0.226	0.320	0.334	0.309	0.296	74.58	74.58	58.91	87.60	42.37
2snw	0.201	0.225	0.244	0.210	0.228	66.14	97.90	83.26	96.06	77.45
3ndf	0.245	0.253	0.258	0.247	0.250	98.72	96.50	44.43	97.19	78.73
5h79	0.299	0.309	0.315	0.306	0.302	69.92	93.28	8.38	91.36	39.53
3m3t	0.244	0.249	0.272	0.254	0.253	59.49	99.22	76.98	96.79	92.82
3hpm	0.299	0.308	0.327	0.292	0.290	44.25	89.78	6.27	80.14	82.58
4bhc	0.251	0.249	0.274	0.258	0.242	71.77	99.21	70.69	99.63	78.36
4c0m	0.316	0.318	0.355	0.317	0.314	32.38	99.91	87.52	100.00	79.74
3k9p	0.283	0.277	0.315	0.260	0.255	88.81	97.71	7.28	96.97	43.24
2jee	0.340	0.404	0.398	0.377	0.332	75.80	87.18	2.15	96.05	16.06
4ug3	0.336	0.277	0.316	0.265	0.278	80.45	98.77	37.12	99.59	91.90
5arj	0.253	0.283	0.349	0.258	0.280	67.66	86.49	4.70	97.98	55.42
2j7i	0.277	0.296	0.318	0.285	0.297	75.38	98.75	32.01	97.73	38.90
6dz6	0.308	0.294	0.303	0.289	0.306	85.62	99.80	43.17	99.50	51.23
2nsb	0.347	0.286	0.292	0.270	0.269	47.58	98.75	97.46	95.30	83.95
5jqz	0.474	0.471	0.610	0.460	0.592	91.86	99.22	37.09	99.22	31.30
